# Migration-dependent extrafollicular programming of preplasmablast age-associated B cells drives lupus pathogenesis

**DOI:** 10.1172/JCI204095

**Published:** 2026-07-14

**Authors:** Taiichiro Shirai, Kentaro Kuzuya, Mizuki Kishi, Shinya Ichikawa, Shuhei Sakakibara, Akiko Nakai, Sarah Leach, Yu-Chen Liu, Daisuke Motooka, Daisuke Okuzaki, Masashi Narazaki, Atsushi Kumanogoh, Tomohiro Kurosaki, Jun Saegusa, Kazuhiro Suzuki

**Affiliations:** 1 Laboratory of Immune Response Dynamics, WPI Immunology Frontier Research Center,; 2 Department of Systems Pharmacology, Graduate School of Medicine, and; 3 Department of Immune Response Dynamics, Research Institute for Microbial Diseases, The University of Osaka, Suita, Osaka, Japan.; 4 Department of Rheumatology and Clinical Immunology, Graduate School of Medicine, Kobe University, Kobe, Hyogo, Japan.; 5 Department of Respiratory Medicine and Clinical Immunology, Graduate School of Medicine and; 6 Laboratory of Systems Immunology, WPI Immunology Frontier Research Center, The University of Osaka, Suita, Osaka, Japan.; 7 Graduate School of Medical Safety Management, Jikei University of Health Care Sciences, Osaka, Osaka, Japan.; 8 Department of Bacteriology I, National Institute of Infectious Diseases, JIHS, Shinjuku, Tokyo, Japan.; 9 Laboratory of Human Immunology (Single Cell Genomics), WPI Immunology Frontier Research Center,; 10 NGS Core Facility, Research Institute for Microbial Diseases,; 11 Department of Infection Metagenomics, Research Institute for Microbial Diseases,; 12 Integrated Frontier Research for Medical Science Division, Institute for Open and Transdisciplinary Research Initiatives,; 13 Center for Infectious Disease Education and Research,; 14 Laboratory of Immunopathology, WPI Immunology Frontier Research Center,; 15 Department of Advanced Clinical and Translational Immunology, Graduate School of Medicine, and; 16 Center for Advanced Modalities and DDS, The University of Osaka, Suita, Osaka, Japan.; 17 Laboratory for Lymphocyte Differentiation, RIKEN Center for Integrative Medical Sciences, Yokohama, Kanagawa, Japan.; 18 Laboratory of Lymphocyte Differentiation, WPI Immunology Frontier Research Center, The University of Osaka, Suita, Osaka, Japan.

**Keywords:** Autoimmunity, Immunology, B cells, Cell migration/adhesion, Lupus

## Abstract

Systemic lupus erythematosus (SLE) is an autoimmune disease characterized by autoantibody production. Extrafollicular (EF) B cell responses contribute to SLE pathogenesis, with age-associated B cells (ABCs) giving rise to autoantibody-secreting plasmablasts (PBs). However, the migratory cues governing this EF trajectory remain unclear. Here, we identify a distinct ABC state with PB precursor characteristics (pre-PB ABCs) and reveal a migration-dependent program underlying their generation. Single-cell analysis of patients with SLE and model mice showed that pre-PB ABCs were enriched in autoreactive clones and poised for PB differentiation. Their frequency correlated with autoantibody titers and disease activity, underscoring their pathogenic relevance. We further demonstrated that the oxysterol receptor EBI2 directed ABCs to EF niches within splenic bridging channels, promoting pre-PB ABC formation and autoreactive PB output. This process depended on the COMMD3/8 complex, a positive regulator of chemoattractant receptor signaling. Beyond EBI2-mediated ABC migration to EF niches, the COMMD3/8 complex was also required for trafficking of autoantibody-secreting cells to the bone marrow and infiltration of ABCs into the kidney. Accordingly, COMMD3/8 complex inhibition ameliorated disease in murine SLE models. These findings define a migration-dependent mechanism driving the EF differentiation of ABCs into autoreactive PBs and shaping the tissue distribution of pathogenic B cells, highlighting this program as a potential therapeutic target in SLE.

## Abstract 2

Migration-dependent extrafollicular programming generates autoreactive pre-plasmablast ABCs in lupus; COMMD3/8 complex inhibition disrupts this axis and ameliorates disease.

## Graphical abstract

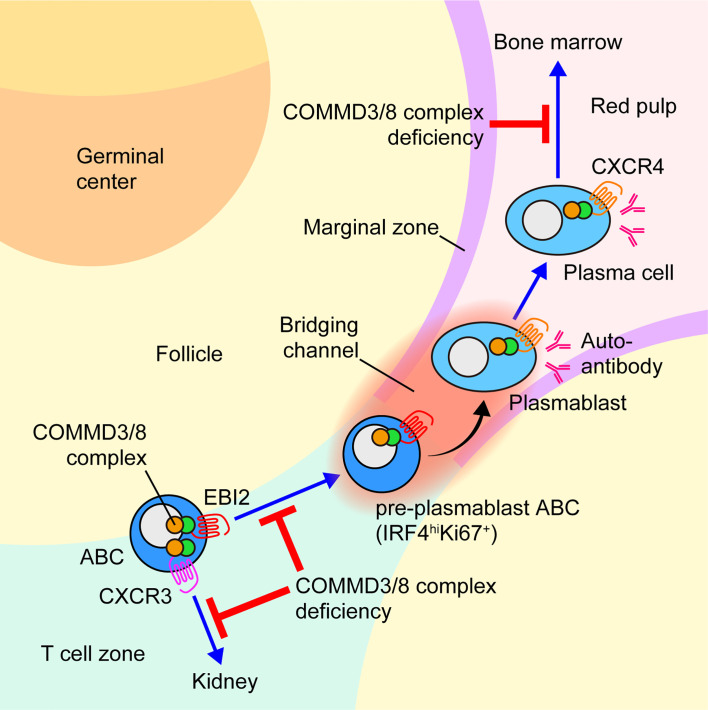

## Introduction

Systemic lupus erythematosus (SLE) is a prototypical systemic autoimmune disease characterized by a breakdown of immune tolerance to nuclear self antigens, leading to the production of autoantibodies and immune complexes that contribute to multiorgan damage, most notably in the kidneys (1). The central role of B cells in SLE pathogenesis is well established (2), as demonstrated by the clinical efficacy of B cell–targeted therapies such as B cell–depleting antibodies and CAR T cells (3, 4). However, these approaches indiscriminately eliminate broad B cell populations, lacking selectivity for pathogenic subsets, and can cause excessive immunosuppression and increased infection susceptibility. These limitations underscore the need to identify pathogenic B cell populations and elucidate their regulatory mechanisms to enable more selective and durable therapies.

Accumulating evidence indicates that age-associated B cells (ABCs) constitute a distinct effector B cell population that expands in patients with SLE and contributes to disease pathogenesis (5, 6). Although initially identified in aged or autoimmune-prone mice (7, 8), human and murine ABCs exhibit overlapping features (9, 10), including expression of CD11c and a requirement for the transcription factor ZEB2 in their development (11). ABCs are enriched for autoreactive specificities and can give rise to autoantibody-secreting plasmablasts (PBs) that further mature into plasma cells (PCs) (5, 9, 10). However, the characteristics of ABCs poised for differentiation into autoreactive PBs remain incompletely understood.

During humoral immune responses, activated B cells commit to either the germinal center (GC) or the extrafollicular (EF) pathway to generate PBs (12, 13). This fate decision is tightly controlled by chemoattractant receptors that regulate B cell migration and positioning within secondary lymphoid organs. In classical antigen-driven responses, B cells entering the GC pathway downregulate the oxysterol receptor Epstein–Barr virus-induced gene 2 (EBI2) and CCR7 while maintaining CXCR5, favoring migration into the follicle interior (14). In contrast, EF-destined B cells retain EBI2 but downregulate CCR7 and CXCR5, enabling migration out of the follicle (14). In SLE, EF responses are suggested to be pathogenic, with ABCs giving rise to autoreactive PBs (5, 15). However, how ABCs are guided to EF niches that support PB generation and what migratory signals govern their positioning remain poorly defined.

We previously identified a protein complex composed of copper metabolism gene MURR1 domain–containing proteins COMMD3 and COMMD8 (the COMMD3/8 complex) as a positive regulator of chemoattractant receptor signaling (16). The COMMD3/8 complex recruits G protein–coupled receptor kinase 6 to ligand-bound receptors, thereby promoting β-arrestin–mediated signaling (16). This complex is also essential for B cell migration during humoral immune responses (16, 17). However, its role in B cell–driven autoimmunity, particularly in the regulation of ABC migration and positioning in SLE, remains unclear.

In this study, we identified a distinct state of ABCs with PB precursor characteristics — termed pre-PB ABCs — enriched in autoreactive clones in both patients with SLE and murine lupus models. We found that EBI2 directs ABC migration to EF foci within splenic bridging channels and that this positioning promotes pre-PB ABC formation and PB responses. Furthermore, we demonstrated that the COMMD3/8 complex facilitates EBI2-mediated ABC localization to EF foci. In addition, it more broadly controlled B cell migratory processes, including antibody-secreting cell (ASC) homing to the bone marrow (BM) and ABC infiltration into the kidney. Inhibition of EBI2 or the COMMD3/8 complex reduced the frequencies of pre-PB ABCs and PBs, accompanied by amelioration of disease. Collectively, our findings uncover a migration-dependent program driving the EF differentiation of ABCs into autoreactive PBs and identify a tractable axis for therapeutic intervention in SLE.

## Results

### Single-cell analysis identifies pre-PB ABCs in human SLE.

**Figure 1:**
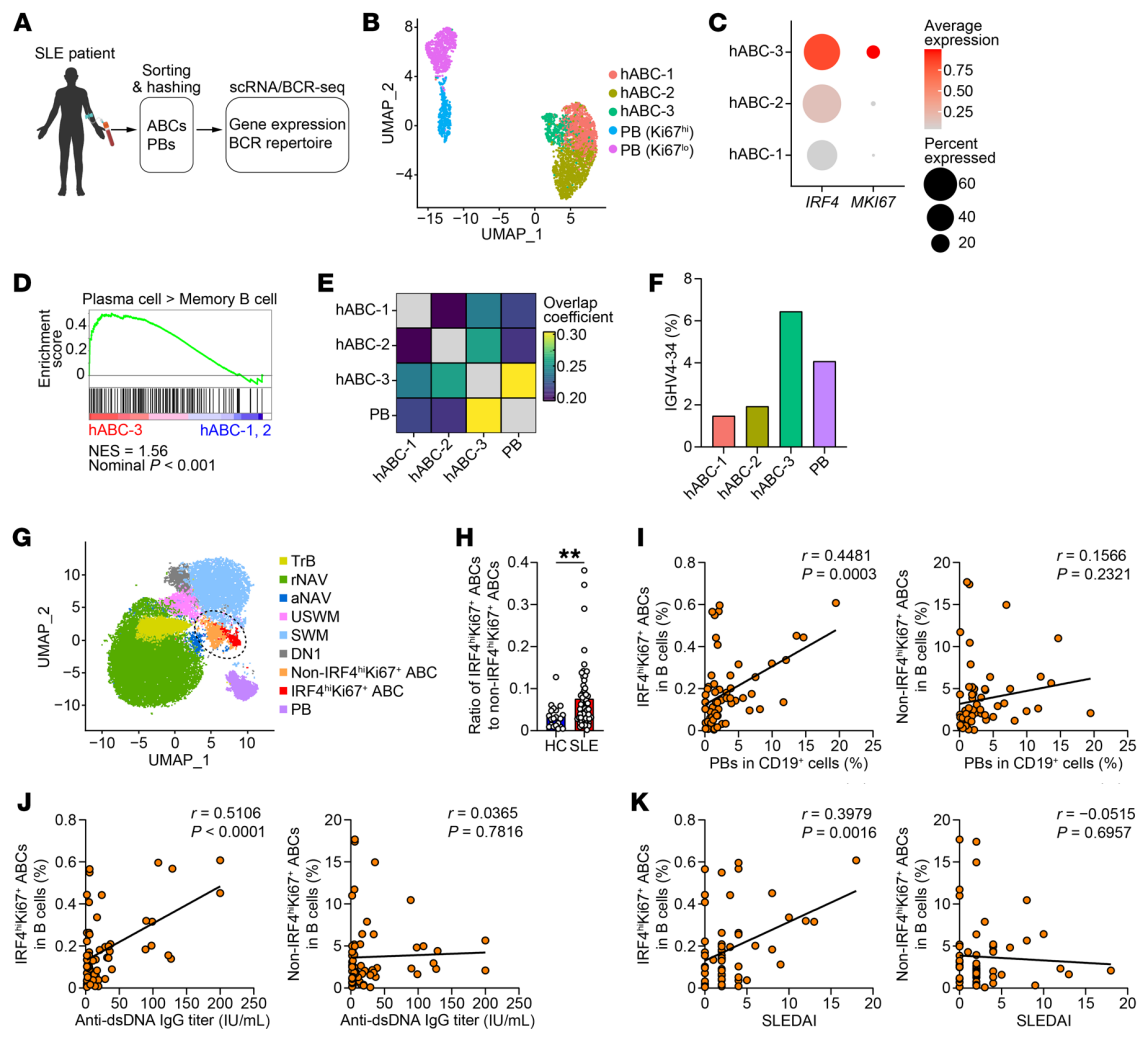
Identification of pre-PB ABCs in patients with SLE. (**A**) Experimental diagram for **B**–**F**. (**B**) UMAP visualization colored by cell type. (**C**) Dot plot showing *IRF4* and *MKI67* expression across cell types. (**D**) GSEA comparing hABC-3 versus hABC-1 plus hABC-2 using the gene set “PLASMA_CELL_VS_MEMORY_BCELL_UP”. NES, normalized enrichment score. (**E**) Heatmap showing normalized clonal overlap among the indicated cell types. (**F**) Proportion of IGHV4-34^+^ IgG^+^ clones in each cell type. (**G**–**K**) Flow cytometry of PBMCs from patients with SLE. (**G**) UMAP visualization of CD19^+^ cells colored by cell type, with ABC clusters outlined. TrB, transitional B cell; rNAV, resting naive B cell; aNAV, activated naive B cell; USWM, unswitched memory B cell; SWM, switched memory B cell. (**H**) Ratio of IRF4^hi^Ki67^+^ to non-IRF4^hi^Ki67^+^ ABC frequencies among B cells (CD19^+^ cells excluding CD27^hi^CD38^hi^ PBs) in people who were healthy controls (HC) and patients. Data are shown as means, with symbols representing individual HC (*n* = 20) or patients (*n* = 60). (**I**–**K**) Correlations of IRF4^hi^Ki67^+^ ABC frequency (left) or non-IRF4^hi^Ki67^+^ ABC frequency (right) with PB frequency (**I**), serum anti-dsDNA IgG titers (**J**), and SLEDAI scores (**K**). Each point represents an individual patient, and the solid line indicates the linear regression fit. The correlation coefficient *r* values were determined by 2-tailed Pearson’s correlation test. *P* values were determined by 2-tailed Mann–Whitney *U* test (**H**) or 2-tailed Pearson’s correlation test (**I**–**K**). **P < 0.01.

We first sought to determine whether human ABCs are heterogeneous and contain a population poised for PB differentiation. To ensure detection of rare subpopulations, ABCs (CD19^+^CD27^–^CD38^–^IgD^–^CD24^–^CD21^lo^CD11c^+^), also referred to as double-negative–2 (DN2) B cells (5), and PBs (CD19^+^CD27^+^CD38^hi^) were sorted from peripheral blood mononuclear cells (PBMCs) of a treatment-naive patient with SLE (Supplemental Table 1; supplemental material available online with this article; https://doi.org/10.1172/JCI204095DS1). Each population was labeled with oligo-tagged hashing antibodies, pooled at a 4:1 ratio, and subjected to single-cell RNA sequencing (scRNA-seq) and single-cell B cell receptor sequencing (scBCR-seq), enabling integrated analysis of their transcriptional states and BCR repertoires (Figure 1A and Supplemental Figure 1A).

Unsupervised clustering identified 3 transcriptionally distinct ABC subpopulations, designated human ABC–1 (hABC-1), hABC-2, and hABC-3 (Figure 1B and Supplemental Figure 1, B and C). Among these, a fraction of hABC-3 showed elevated expression of *IRF4*, a transcription factor essential for PB differentiation, together with *MKI67* (encoding Ki67), consistent with a proliferative phenotype associated with PB differentiation (18, 19) (Figure 1C). Accordingly, gene set enrichment analysis (GSEA) demonstrated enrichment of a PC-associated gene signature in hABC-3 compared with hABC-1 and hABC-2 (Figure 1D). BCR repertoire analysis revealed greater clonal overlap between PBs and hABC-3 than between PBs and either hABC-1 or hABC-2 (Figure 1E), suggesting that hABC-3 is enriched in clones contributing to PB formation. To assess autoreactive potential, we analyzed usage of the immunoglobulin heavy-chain variable region gene (IGHV) 4-34 among IgG^+^ ABCs, because of its well-established association with autoreactivity in human B cell clones (20). Notably, hABC-3 exhibited the highest frequency of IGHV4-34–expressing clones among the ABC subpopulations (Figure 1F). PBs showed a higher frequency than hABC-1 and hABC-2 but a lower frequency than hABC-3. This may reflect PBs that arise directly from activated B cells without passing through the ABC stage (21, 22), partially diluting IGHV4-34 enrichment in bulk PBs. Together, these features suggest that hABC-3 contains cells with PB precursor characteristics and enrichment of autoreactive clones, which we refer to as pre-PB ABCs.

To examine the frequency and expansion of pre-PB ABCs across individuals with SLE (Supplemental Table 2), we performed high-dimensional flow cytometry on patient-derived PBMCs. Consistent with the scRNA-seq findings, we identified IRF4^hi^Ki67^+^ ABCs within the ABC compartment, defined by relatively higher IRF4 expression among ABCs (Figure 1G and Supplemental Figure 1A). This population likely corresponds to pre-PB ABCs. Both IRF4^hi^Ki67^+^ ABCs and non-IRF4^hi^Ki67^+^ ABCs were increased in patients with SLE compared with healthy individuals (Supplemental Figure 1D). Importantly, the relative enrichment of IRF4^hi^Ki67^+^ ABCs over non-IRF4^hi^Ki67^+^ ABCs was significantly greater in patients with SLE, suggesting preferential expansion of the former (Figure 1H). The frequency of IRF4^hi^Ki67^+^ ABCs among B cells positively correlated with PB frequency (Figure 1I) and serum anti-dsDNA IgG titers (Figure 1J), but not total IgG titers (Supplemental Figure 1E). These cells also exhibited negative correlations with serum complement components C3 and C4 (Supplemental Figure 1, F and G). Notably, their frequency positively correlated with disease severity, as measured by the SLE disease activity index (SLEDAI) scores (Figure 1K). In contrast, non-IRF4^hi^Ki67^+^ ABCs showed no significant correlations with any of these parameters (Figure 1, I–K, and Supplemental Figure 1, E–G). Thus, IRF4^hi^Ki67^+^ ABCs, unlike other ABC populations, are preferentially associated with PB output, autoantibody production, and clinical disease activity in patients with SLE.

Collectively, these findings identify IRF4^hi^Ki67^+^ ABCs as a distinct autoreactive ABC state with pre-PB features and highlight their disease relevance in SLE.

### A murine lupus model reveals a conserved pre-PB ABC state.

Having identified pre-PB ABCs in patients with SLE, we next asked whether a comparable population exists in mice and sought to define its phenotypic and functional properties. Because human ABC studies rely primarily on PBMCs and offer limited access to lymphoid tissues where autoantibody responses occur, murine lupus models provide a valuable system to characterize pre-PB ABCs in vivo. Enhanced TLR7 signaling drives lupus pathology and promotes ABC expansion in both humans and mice (9, 10, 23). Furthermore, a recent study identified a gain-of-function *TLR7* mutation as a monogenic cause of human SLE characterized by marked ABC accumulation (15). Therefore, we employed a murine model in which lupus-like pathology was induced by chronic TLR7 stimulation (11, 24).

We first sought to determine the relative contributions of GC and EF responses to the generation of ABCs and PBs in this lupus model. To this end, we utilized *S1pr2^CreERT2/+^Rosa26^loxP-STOP-loxP-tdTomato/+^* (*S1pr2*-tdTomato) mice, in which tamoxifen-induced Cre recombination permanently labels GC B cells and their progeny with tdTomato (25). *S1pr2*-tdTomato mice were treated with the TLR7 agonist resiquimod (RSQ) and administered tamoxifen starting 1 week after the initiation of RSQ treatment (Supplemental Figure 2A). After an additional 3 weeks of RSQ treatment, we assessed tdTomato labeling by flow cytometry in GC B cells (CD19^+^IgD^lo^CD95^+^GL7^+^CD38^–^) (25), ABCs (B220^+^CD19^+^CD138^–^CD11b^+^CD11c^+^) (26), and PBs (B220^+^CD19^+^CD138^+^CXCR3^+^MHC-II^hi^) (27). Fewer than 10% of ABCs, as well as a comparable proportion of PBs, were labeled with tdTomato, despite almost complete labeling of GC B cells (Supplemental Figure 2, B and C). Thus, most ABCs and PBs arise independently of GC responses in the TLR7-induced lupus model.

**Figure 2:**
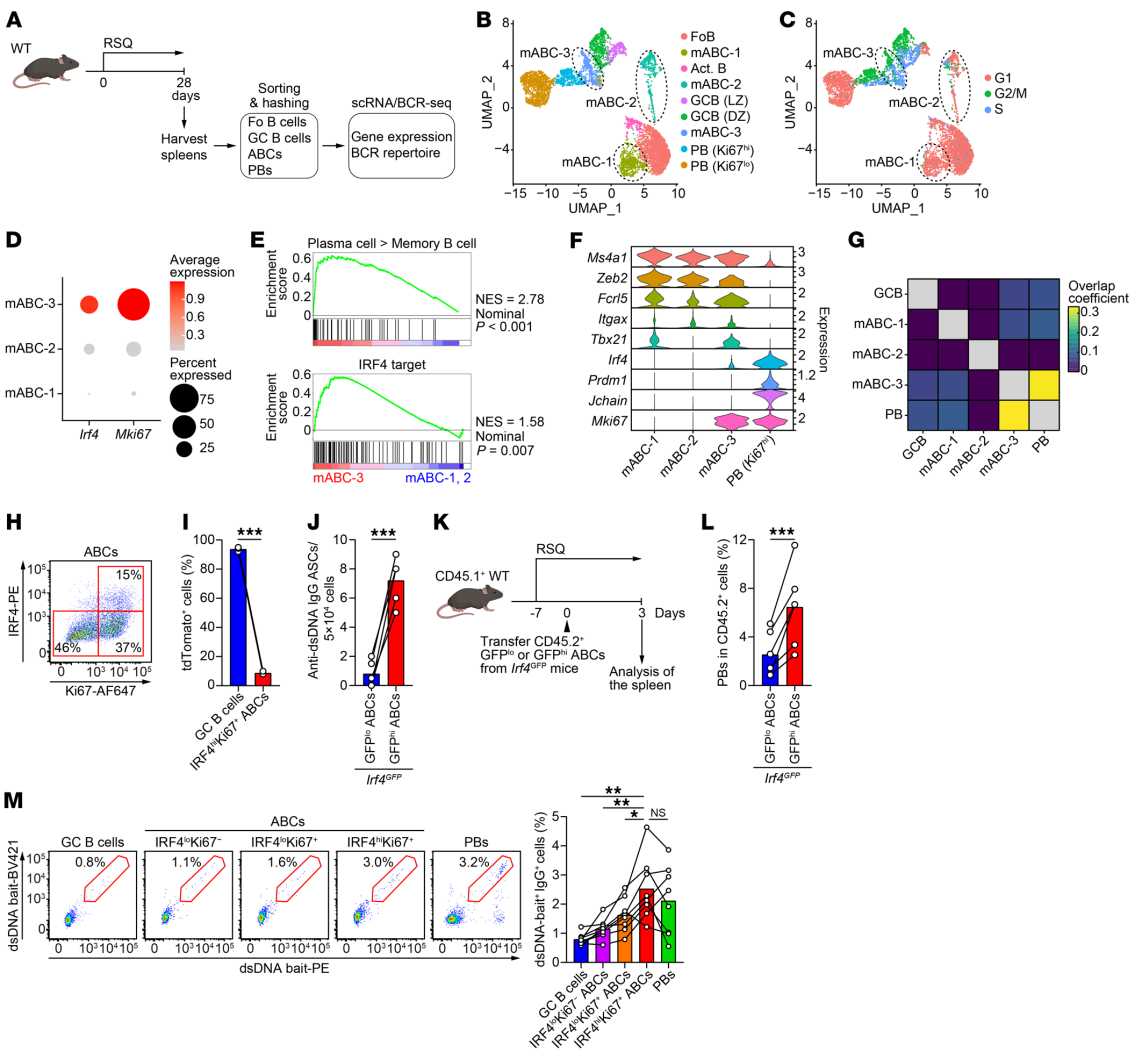
Identification of pre-PB ABCs in TLR7-induced lupus. (**A**) Experimental diagram for **B**–**G**. Spleens were harvested from 2 mice. (**B** and **C**) UMAP visualization colored by cell type (**B**) or cell-cycle phase (**C**), with ABC clusters outlined. Act. B, activated B cell; LZ, light zone; DZ, dark zone. (**D**) Dot plot showing *Irf4* and *Mki67* expression across cell types. (**E**) GSEA comparing mABC-3 versus mABC-1 plus mABC-2 using the gene sets “PLASMA_CELL_VS_MEMORY_BCELL_UP” (top) and “IRF4_TARGETS” (bottom). (**F**) Violin plots showing the expression levels of the indicated genes across cell types. (**G**) Heatmap showing normalized clonal overlap among the indicated cell types. (**H**) Flow cytometry plot showing IRF4 and Ki67 expression in ABCs from the spleens of RSQ-treated WT mice. (**I**) Frequencies of tdTomato^+^ cells in GC B cells and IRF4^hi^Ki67^+^ ABCs from the spleens of *S1pr2*-tdTomato mice, analyzed as in Supplemental Figure 2A. (**J**) Numbers of anti-dsDNA IgG ASCs generated in vitro from GFP^lo^ and GFP^hi^ ABCs isolated from RSQ-treated *Irf4^GFP^* mice, measured by an enzyme-linked immunosorbent spot (ELISPOT) assay. (**K** and **L**) PB differentiation from adoptively transferred GFP^lo^ or GFP^hi^ ABCs isolated from RSQ-treated *Irf4^GFP^* mice. PB frequencies among donor-derived (CD45.2^+^) cells were determined by flow cytometry. (**M**) Frequencies of dsDNA bait^+^ cells among IgG^+^ cells in the indicated B-lineage populations from the spleens of RSQ-treated WT mice. Representative flow cytometry plots are shown. Data are shown as means, with symbols representing individual mice (**I**, **J**, **L**, and **M**). Results are representative of 2 independent experiments. *P* values were determined by 2-tailed paired *t* test (**I**, **J**, and **L**) or 1-way ANOVA with Dunnett’s post hoc test (**M**). *P < 0.05; **P < 0.01; ***P < 0.001.

We next aimed to clarify how ABCs relate to other B-lineage subsets, including PBs, in TLR7-induced lupus at single-cell resolution. Follicular (Fo) B cells (B220^+^CD19^+^CD93^–^CD21^int^CD23^hi^), GC B cells, ABCs, and PBs were sorted from the spleens of RSQ-treated WT mice (Supplemental Figure 2C). Each population was labeled with oligo-tagged hashing antibodies, pooled in equal numbers, and subjected to scRNA/BCR-seq (Figure 2A). Transcriptional clustering resolved ABCs into 3 distinct subpopulations, designated murine ABC (mABC)-1, mABC-2, and mABC-3 (Figure 2B and Supplemental Figure 2, D and E). Among these, mABC-3 exhibited the highest proliferative activity, as determined by cell-cycle analysis (Figure 2C), in line with pathway enrichment for cell division (Supplemental Figure 2F). mABC-1 was enriched for the MHC class II antigen presentation pathway, consistent with conventional ABC features (28), whereas mABC-2 exhibited low expression of a canonical ABC marker gene *Tbx21* and was associated with complement-related pathways (Supplemental Figure 2, E and F).

Among the ABC subpopulations, a fraction of mABC-3 showed high expression of *Irf4* and *Mki67*, distinguishing it from other ABC subpopulations (Figure 2D). GSEA further revealed enrichment of PC-associated gene signatures in mABC-3 relative to mABC-1 and mABC-2 (Figure 2E). mABC-3 retained expression of canonical ABC marker genes, including *Ms4a1*, *Zeb2*, *Fcrl5*, *Itgax*, and *Tbx21* (11) (Figure 2F). In contrast, it lacked expression of PB-defining genes such as *Prdm1* (encoding Blimp-1) and *Jchain*, and its *Irf4* levels remained lower than those in PBs. These features suggest that mABC-3 does not represent PBs but likely corresponds with a precursor state along the PB differentiation pathway. Supporting this, BCR repertoire analysis revealed greater clonal overlap between PBs and mABC-3 than between PBs and mABC-1, mABC-2, or GC B cells (Figure 2G), suggesting that mABC-3 is enriched in clones with PB-differentiation potential. Together, these transcriptional and clonal features suggest that mABC-3 contains a pre-PB ABC population analogous to that in human SLE.

Consistent with the scRNA-seq results, flow cytometry identified IRF4^hi^Ki67^+^ ABCs within the splenic ABC compartment of RSQ-treated mice (Figure 2H). These cells likely correspond with the proliferative population of mABC-3 with high *Irf4* expression and may represent pre-PB ABCs. In contrast, IRF4^lo^Ki67^+^ ABCs likely correspond to a proliferative population of mABC-3 with low *Irf4* expression, whereas IRF4^lo^Ki67^–^ ABCs appear to comprise a mixed population of mABC-1 and mABC-2. We confirmed that IRF4^hi^Ki67^+^ ABCs exhibited lower IRF4 levels and minimal *Prdm1* expression compared with PBs (Supplemental Figure 2, G and H). To define the developmental origin of pre-PB ABCs, we analyzed IRF4^hi^Ki67^+^ ABCs in RSQ-treated *S1pr2*-tdTomato mice and found that fewer than 10% were labeled with tdTomato (Figure 2I). This result suggests that pre-PB ABCs are generated predominantly in a GC-independent manner.

To functionally validate the PB differentiation potential of pre-PB ABCs, GFP^lo^ and GFP^hi^ ABCs (corresponding to IRF4^lo^ and IRF4^hi^ ABCs, respectively) were sorted from the spleens of RSQ-treated *Irf4^GFP^* mice (Supplemental Figure 2, I and J). These cells were cultured under RSQ stimulation and analyzed for the generation of anti-dsDNA IgG ASCs. Compared with GFP^lo^ ABCs, GFP^hi^ ABCs gave rise to higher numbers of anti-dsDNA IgG ASCs (Figure 2J). To assess the precursor-product relationship in vivo, these cells were adoptively transferred into congenically distinct RSQ-treated recipients (Figure 2K). Consistent with the in vitro findings, GFP^hi^ ABCs preferentially differentiated into PBs (Figure 2L and Supplemental Figure 2K), suggesting that IRF4^hi^ ABCs have an enhanced capacity for PB differentiation. Flow cytometry using fluorophore-conjugated dsDNA as bait revealed that IRF4^hi^Ki67^+^ ABCs exhibited a higher frequency of dsDNA-binding IgG^+^ cells, comparable with that in PBs and exceeding that in GC B cells and other ABC populations (Figure 2M and Supplemental Figure 2, L–N). Together, these findings suggest that pre-PB ABCs are poised for PB differentiation and exhibit increased autoreactivity compared with other ABC populations.

Collectively, these findings support the idea that IRF4^hi^Ki67^+^ ABCs represent a conserved autoreactive ABC state with pre-PB features in both humans and mice and suggest that they may serve as a source of autoantibody-secreting PBs in SLE pathogenesis.

### EBI2-mediated ABC migration to EF niches promotes pre-PB ABC formation.

Next, we investigated the mechanisms driving pre-PB ABC formation. During EF responses, the marginal zone bridging channels in the spleen, where the T cell zone abuts the red pulp, are thought to serve as a specialized EF niche that supports PB generation (12, 21, 29). Within these regions, type-2 conventional dendritic cells (cDC2s) are considered to provide trophic factors such as B cell–activating factor (BAFF) and a proliferation-inducing ligand (APRIL) that promote the survival of differentiating PBs (30–32). Notably, ligands for the chemoattractant receptor EBI2 are abundant in the bridging channels (33). These observations led us to hypothesize that EBI2 guides ABCs into EF niches, thereby promoting pre-PB ABC formation.

**Figure 3:**
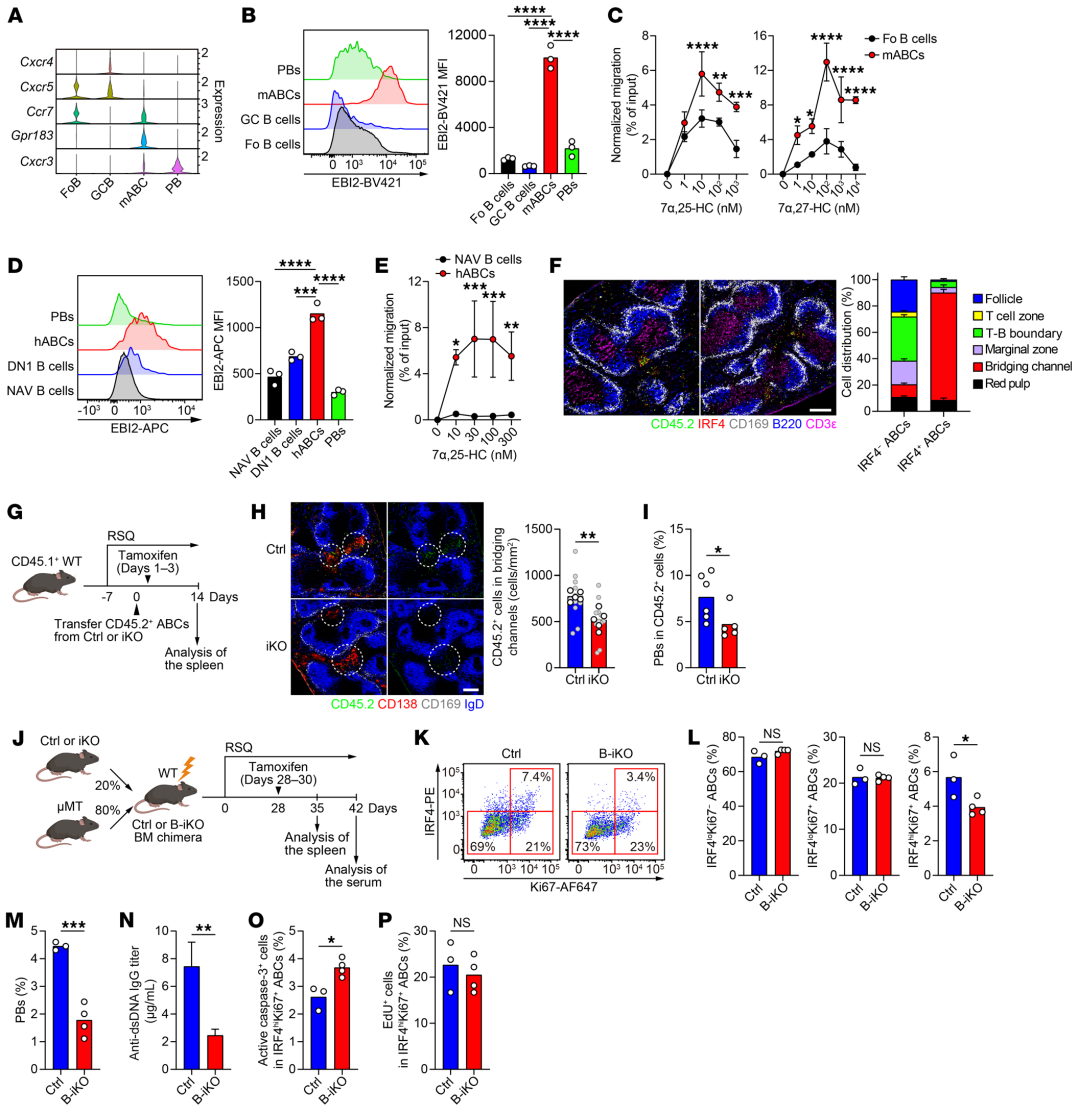
Requirement for EBI2 in ABC migration to EF niches and pre-PB ABC formation. (**A**) Expression of the indicated genes in B-lineage cells. (**B**) Flow cytometry of EBI2 expression on B-lineage cells. (**C**) Transwell migration of splenic Fo B cells and ABCs from RSQ-treated WT mice in response to the EBI2 ligands. Data are shown as mean ± SD of technical triplicates. (**D**) Flow cytometry of EBI2 expression on B-lineage cells from patients with SLE. (**E**) Transwell migration of naive B cells and ABCs from patients with SLE in response to the EBI2 ligand. Data are shown as mean ± SD of 3 independent donors. (**F**) Splenic distribution of CD45.2^+^ ABCs from RSQ-treated WT mice after transfer into CD45.1^+^ WT recipients. Representative immunofluorescence images are shown (scale bar: 200 μm). Percentages of CD45.2^+^ cells in the indicated regions were quantified in 15 sections from 5 spleens. Data are shown as mean + SEM. (**G**) Experimental diagram for **H** and **I**. CD45.2^+^ ABCs from *Rosa26^CreERT2/+^Gpr183^fl/+^* (control, Ctrl) or *Rosa26^CreERT2/+^Gpr183^fl/fl^* (inducible knockout, iKO) mice were transferred into RSQ-treated CD45.1^+^ WT recipients and analyzed after tamoxifen administration. (**H**) Splenic distribution of CD45.2^+^ cells. Representative images are shown with bridging channels circled (scale bar: 200 μm). (**I**) Frequency of PBs in CD45.2^+^ cells. (**J**) Experimental diagram for **K**–**P**. BM chimeras were generated using μMT BM plus *Rosa26^CreERT2/+^Gpr183^fl/+^* BM (Ctrl) or *Rosa26^CreERT2/+^Gpr183^fl/fl^* BM (B cell-specific inducible knockout, B-iKO). RSQ-treated chimeras were administered tamoxifen and analyzed. (**K**) Flow cytometry of ABCs. (**L** and **M**) Frequencies of ABC populations in total ABCs (**L**) and of PBs in splenocytes (**M**). (**N**) Serum anti-dsDNA IgG titers measured by ELISA. Data are shown as mean + SEM (*n* = 10 Ctrl, 11 B-iKO). (**O** and **P**) Frequencies of active caspase-3^+^ (**O**) and EdU^+^ (**P**) cells in IRF4^hi^Ki67^+^ ABCs. Data are shown as means, with symbols representing individual mice (**B**, **H**, **I**, **L**, **M**, **O**, and **P**) or patients (**D**). In (**H**), gray symbols represent individual bridging channels. In **B** and **D**, representative histograms and MFI are shown. Results are representative of 2 independent experiments. *P* values were determined by 1-way ANOVA with Dunnett’s post hoc test (**B** and **D**), 2-way ANOVA with Šídák’s post hoc test (**C** and **E**), or 2-tailed unpaired *t* test (**H**, **I**, and **L**–**P**). *P < 0.05; **P < 0.01; ***P < 0.001; ****P < 0.0001.

To test this hypothesis, we examined chemoattractant receptor expression in ABCs. Transcriptomic analysis of the scRNA-seq dataset shown in Figure 2 revealed upregulation of *Gpr183* (encoding EBI2) in ABCs relative to Fo B cells, GC B cells, and PBs, whereas *Cxcr5* was downregulated (Figure 3A). Surface EBI2 expression was also elevated on ABCs (Figure 3B and Supplemental Figure 3A). Functionally, ABCs exhibited enhanced chemotactic responses toward the EBI2 ligands, 7α,25-dihydroxycholesterol (7α,25-HC) and 7α,27-HC, compared with Fo B cells (Figure 3C). Consistently, in human peripheral blood B–lineage cells, surface EBI2 expression was higher on ABCs than on naive B cells, DN1 B cells (which typically express CXCR5 and are thought to arise from GC responses) (5), and PBs (Figure 3D). Human ABCs also showed a greater chemotactic response toward the EBI2 ligand than naive B cells (Figure 3E). These findings suggest that EBI2 plays an important role in directing ABC migration in both mice and humans.

To determine the distribution of ABCs in the spleen, we adoptively transferred ABCs from RSQ-treated mice into congenically distinct recipients. Immunofluorescence analysis revealed that ABCs with undetectable IRF4 (IRF4^–^ ABCs) were mainly located at the T-B boundary, within follicles, and in the marginal zone (Figure 3F and Supplemental Figure 3B). In contrast, IRF4^+^ ABCs were preferentially localized to bridging channels (Figure 3F and Supplemental Figure 3B), suggesting that pre-PB ABCs are enriched in EF foci within bridging channels. At this time point, the transferred ABCs had not yet differentiated into PBs, as confirmed by flow cytometry (Supplemental Figure 3C).

To assess whether EBI2 is involved in the EF localization of pre-PB ABCs, we adoptively transferred ABCs from RSQ-treated *Rosa26^CreERT2/+^Gpr183^fl/fl^* mice into congenically distinct RSQ-treated recipients. The recipient mice were then administered tamoxifen to induce Cre-mediated deletion of *Gpr183* in the transferred cells (Figure 3G). Immunofluorescence analysis revealed that EBI2-deficient ABCs showed reduced localization to the EF foci within the bridging channels (Figure 3H). This was accompanied by decreased PB differentiation (Figure 3I). These findings suggest that EBI2 directs ABCs to EF niches, a process that may facilitate the formation of pre-PB ABCs and their differentiation into PBs.

To examine the role of EBI2 in the maintenance of pre-PB ABCs under chronic TLR7 stimulation, we generated mixed BM chimeras in which *Gpr183* deletion could be induced specifically in B cells. BM cells from μMT mice, which lack mature B cells, were mixed in a 4:1 ratio with *Rosa26^CreERT2/+^Gpr183^fl/fl^* BM cells and transferred into irradiated hosts. Tamoxifen administration after 4 weeks of RSQ treatment selectively reduced the frequency of IRF4^hi^Ki67^+^ ABCs, whereas IRF4^lo^Ki67^–^ and IRF4^lo^Ki67^+^ ABCs were unaffected (Figure 3, J–L). The reduction in IRF4^hi^Ki67^+^ ABCs was associated with impaired PB generation and decreased serum anti-dsDNA IgG titers (Figure 3, M and N). Mechanistically, EBI2 deficiency led to enhanced apoptosis of IRF4^hi^Ki67^+^ ABCs without affecting their proliferation (Figure 3, O and P). These results suggest that EBI2 supports the maintenance of pre-PB ABCs under sustained TLR7 stimulation, at least in part, by promoting their survival.

We found that IRF4^+^ ABCs were positioned in close proximity to cDC2s within the bridging channels (Supplemental Figure 3D). While BAFF receptor expression was comparable among ABC populations (Supplemental Figure 3E), the expression of TACI, a receptor for both BAFF and APRIL, was upregulated on IRF4^hi^Ki67^+^ ABCs (Supplemental Figure 3F). To assess the impact of cDC2-derived soluble trophic factors, we performed a noncontact coculture of ABCs with cDC2s (Supplemental Figure 3G). This coculture increased the proportion of IRF4^hi^Ki67^+^ ABCs and suppressed their apoptosis (Supplemental Figure 3, H and I). These effects were abrogated by the addition of a TACI-Fc fusion protein. These findings suggest that cDC2-derived, TACI-mediated signals may provide survival cues to pre-PB ABCs.

Taken together, these observations suggest that EBI2-mediated positioning of ABCs into EF niches within bridging channels promotes pre-PB ABC formation, which may contribute to autoreactive PB output in lupus.

### B cell–specific EBI2 deficiency mitigates lupus pathology.

**Figure 4:**
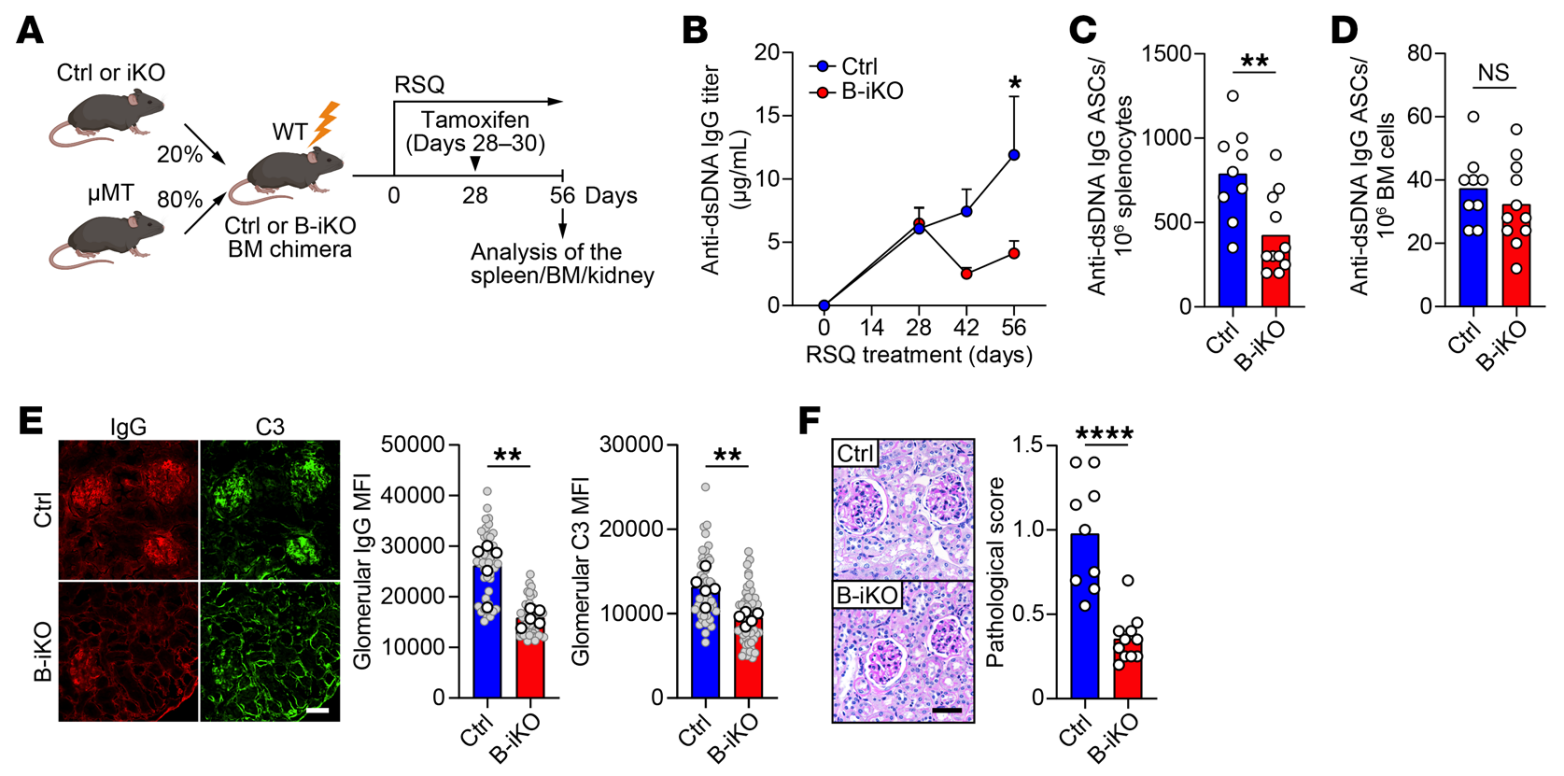
Effects of B cell–specific EBI2 deficiency on lupus pathology. (**A**) Experimental diagram. Ctrl and B-iKO BM chimeras generated as in Figure 3J were treated with RSQ and administered tamoxifen. (**B**) Serum anti-dsDNA IgG titers measured by ELISA. Data are shown as mean + SEM (*n* = 10 Ctrl, 11 B-iKO). (**C** and **D**) Anti-dsDNA IgG ASC numbers in the spleen (**C**) and BM (**D**) analyzed by ELISPOT. (**E**) IgG and C3 deposits in the renal glomeruli analyzed by immunofluorescence microscopy. MFI of IgG and C3 was quantified. (**F**) Periodic acid–Schiff–stained (PAS-stained) glomerular sections and pathological scores. Data are shown as means, with symbols representing individual mice (**C**–**F**). In **E**, gray symbols represent individual glomeruli. In **E** and **F**, representative images are shown (scale bar: 50 μm). Results are representative of 2 independent experiments. *P* values were determined by 2-way ANOVA with Šídák’s post hoc test (**B**) or 2-tailed unpaired *t* test (**C**–**F**). *P < 0.05; **P < 0.01; ****P < 0.0001.

Because EBI2-mediated ABC migration promotes the EF generation of autoreactive PBs, we investigated the B cell–intrinsic role of EBI2 in lupus pathology. To selectively delete *Gpr183* in B cells, we used the mixed BM chimeras generated with BM cells from μMT and *Rosa26^CreERT2/+^Gpr183^fl/fl^* mice (Figure 4A). Tamoxifen administration after 4 weeks of RSQ treatment decreased serum anti-dsDNA IgG titers (Figure 4B). The number of anti-dsDNA IgG ASCs was reduced in the spleen but unaffected in the BM (Figure 4, C and D). Accordingly, nephritis was ameliorated, with diminished immune complex deposition in glomeruli and improved glomerular pathology (Figure 4, E and F). Together, these findings suggest that B cell–expressed EBI2 promotes lupus pathology.

### The COMMD3/8 complex promotes EBI2-dependent ABC migration and pre-PB ABC formation.

**Figure 5:**
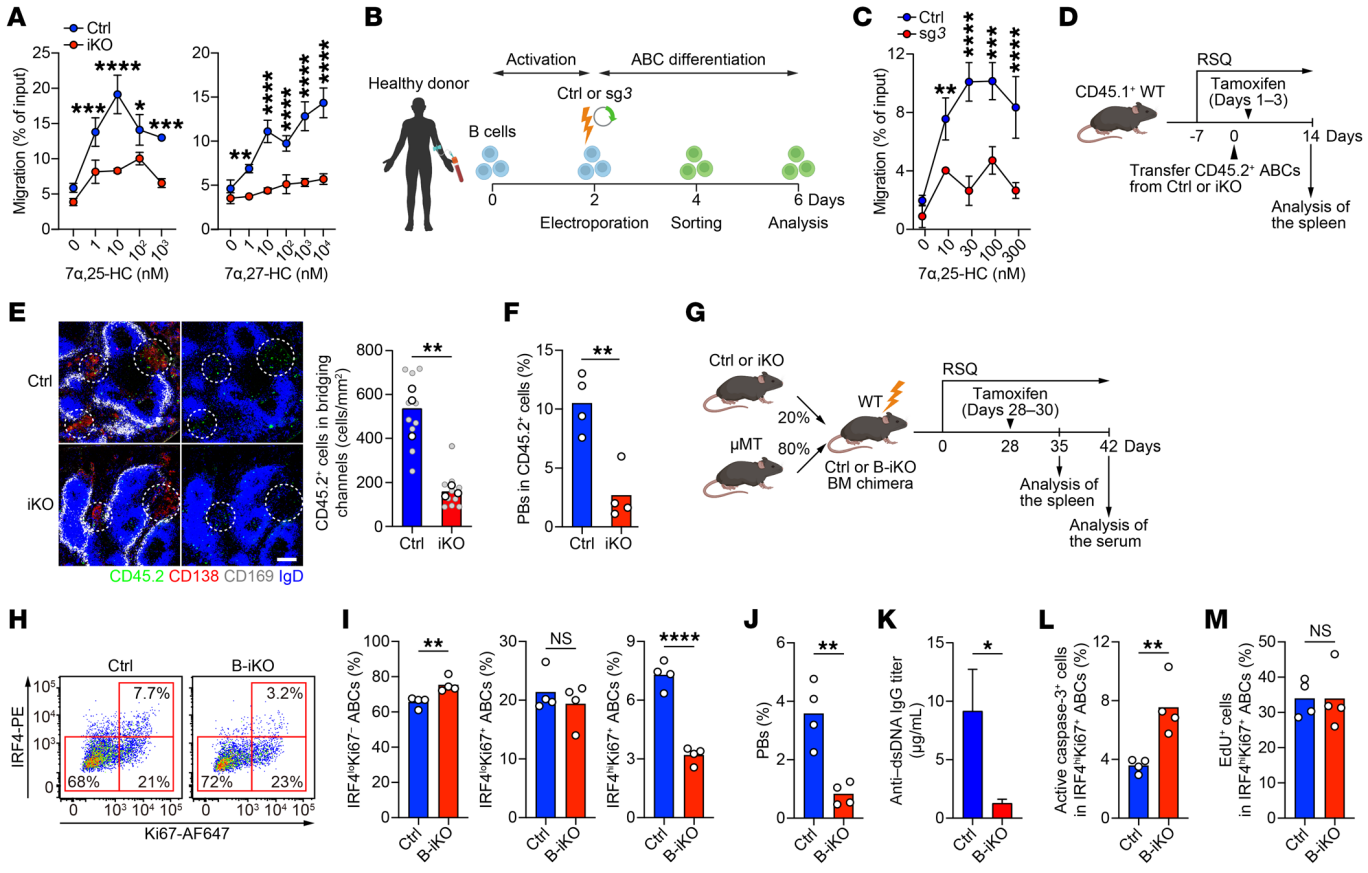
Role of the COMMD3/8 complex in EBI2-dependent ABC migration and pre-PB ABC formation. (**A**) Transwell migration of splenic ABCs from RSQ-treated *Rosa26^CreERT2/+^Commd3^fl/+^* (Ctrl) and *Rosa26^CreERT2/+^Commd3^fl/fl^* (iKO) mice in response to the EBI2 ligands. Data are shown as mean ± SD of technical triplicates. (**B**) Experimental diagram for **C**. B cells from healthy donors were differentiated into ABCs, transfected with a CRISPR/Cas9 plasmid containing no sgRNA (Ctrl) or a *COMMD3*-targeting sgRNA (sg*3*), and subjected to migration assays. (**C**) Transwell migration of human ABCs in response to the EBI2 ligand. Data are shown as mean ± SD of technical triplicates. (**D**) Experimental diagram for **E** and **F**. CD45.2^+^ ABCs from Ctrl or iKO mice were transferred into RSQ-treated CD45.1^+^ WT recipients and analyzed after tamoxifen administration. (**E**) Splenic distribution of CD45.2^+^ cells. Representative images are shown with bridging channels circled (scale bar: 200 μm). (**F**) Frequency of PBs in CD45.2^+^ cells. (**G**) Experimental diagram for **H**–**M**. BM chimeras were generated using μMT BM plus *Rosa26^CreERT2/+^Commd3^fl/+^* BM (Ctrl) or *Rosa26^CreERT2/+^Commd3^fl/fl^* BM (B-iKO). RSQ-treated chimeras were administered tamoxifen and analyzed. (**H**) Flow cytometry of ABCs. (**I** and **J**) Frequencies of ABC populations in total ABCs (**I**) and of PBs in splenocytes (**J**). (**K**) Serum anti-dsDNA IgG titers measured by ELISA. Data are shown as mean + SEM (*n* = 6 Ctrl, 7 B-iKO). (**L** and **M**) Frequencies of active caspase-3^+^ (**L**) and EdU^+^ (**M**) cells in IRF4^hi^Ki67^+^ ABCs. Data are shown as means, with symbols representing individual mice (**E**, **F**, **I**, **J**, **L**, and **M**). In **E**, gray symbols represent individual bridging channels. Results are representative of 2 independent experiments. *P* values were determined by 2-way ANOVA with Šídák’s post hoc test (**A** and **C**) or 2-tailed unpaired *t* test (**E**, **F**, and **I**–**M**). *P < 0.05; **P < 0.01; ***P < 0.001; ****P < 0.0001.

Building on the role of EBI2-mediated migration in pre-PB ABC formation, we next investigated the involvement of the COMMD3/8 complex in this process, as it positively regulates chemoattractant receptor signaling (16). We used *Rosa26^CreERT2/+^Commd3^fl/fl^* mice, in which tamoxifen administration induces the loss of both COMMD3 and COMMD8 proteins due to their mutual dependence for stability (16, 17). COMMD3/8-complex deficiency strongly suppressed chemotactic responses of ABCs toward EBI2 ligands (Figure 5A). Consistently, EBI2-dependent chemotaxis of in vitro–differentiated human ABCs was also impaired under COMMD3/8 complex–deficient conditions (Figure 5, B and C, and Supplemental Figure 4, A and B). Thus, the COMMD3/8 complex is required for EBI2-mediated ABC migration in both mice and humans.

To determine whether COMMD3/8-complex deficiency affects ABC localization to EF niches, we adoptively transferred ABCs from RSQ-treated *Rosa26^CreERT2/+^Commd3^fl/fl^* mice into congenically distinct RSQ-treated recipients, followed by tamoxifen administration (Figure 5D). Recapitulating the EBI2-deficient phenotype, COMMD3/8 complex–deficient ABCs showed decreased localization to the EF foci within the bridging channels (Figure 5E), accompanied by reduced PB generation (Figure 5F). These results suggest that the COMMD3/8 complex is required for EBI2-mediated ABC positioning in EF niches that support PB output.

To examine the contribution of the COMMD3/8 complex to the maintenance of pre-PB ABCs under chronic TLR7 stimulation, we generated mixed BM chimeras using BM cells from μMT and *Rosa26^CreERT2/+^Commd3^fl/fl^* mice, enabling inducible B cell–specific deletion of *Commd3*. Induction of COMMD3/8-complex deficiency after 4 weeks of RSQ treatment selectively reduced IRF4^hi^Ki67^+^ ABCs among the ABC populations (Figure 5, G–I). This was associated with impaired PB generation and decreased anti-dsDNA IgG production (Figure 5, J and K). COMMD3/8-complex deficiency increased apoptosis of IRF4^hi^Ki67^+^ ABCs without affecting their proliferation (Figure 5, L and M). Consistently, scRNA-seq analysis revealed a selective reduction of mABC-3 in the absence of the COMMD3/8 complex (Supplemental Figure 4, C–E). GSEA showed enrichment of an apoptosis-related gene signature, with no transcriptional evidence of altered TLR or BCR signaling in COMMD3/8 complex–deficient mABC-3 (Supplemental Figure 4F).

Importantly, COMMD3/8-complex deficiency did not impair in vitro differentiation of B cells into ABCs and PBs following stimulation with RSQ, anti-IgM antibodies, and IFN-γ (Supplemental Figure 4, G and H). In addition, TLR7 and EBI2 expression levels were preserved in COMMD3/8 complex–deficient ABCs (Supplemental Figure 4, I and J). Their antigen-presenting capacity — which can contribute to lupus pathogenesis (34, 35) — also remained intact (Supplemental Figure 4K). Taken together, these findings suggest that the COMMD3/8 complex promotes EF generation of PBs by supporting the migration-dependent, niche-restricted survival of pre-PB ABCs.

### COMMD3/8-complex deficiency ameliorates lupus pathology.

Given that the COMMD3/8 complex promotes the generation of ABC-derived PBs during EF responses, we investigated its role in disease progression in TLR7-induced lupus. To inactivate the COMMD3/8 complex after disease onset, we administered tamoxifen to *Rosa26^CreERT2/+^Commd3^fl/fl^* mice after 4 weeks of RSQ treatment, when anti-dsDNA antibodies were detectable in the serum (Supplemental Figure 5A). Systemic COMMD3/8-complex deficiency suppressed anti-dsDNA IgG titers and reduced anti-dsDNA IgG ASC numbers in both the spleen and BM (Supplemental Figure 5, B–D). Accordingly, glomerular pathology was ameliorated and survival was improved (Supplemental Figure 5, E–G). These findings suggest that the COMMD3/8 complex plays an important role in disease progression in this lupus model.

**Figure 6:**
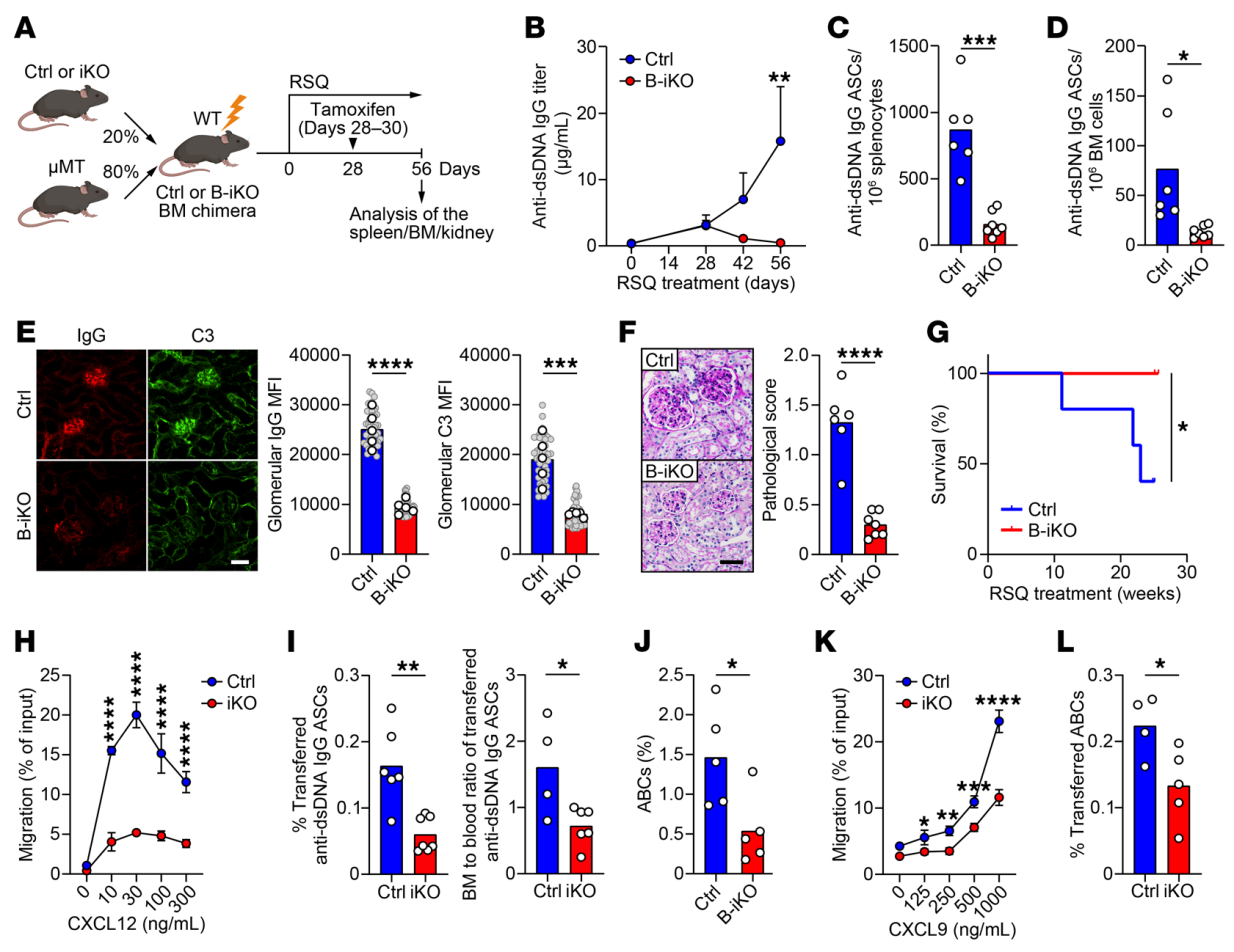
Amelioration of lupus pathology by COMMD3/8-complex deficiency. (**A**) Experimental diagram for **B**–**G**. Ctrl and B-iKO BM chimeras generated as in Figure 5G were treated with RSQ and administered tamoxifen. (**B**) Serum anti-dsDNA IgG titers measured by ELISA. Data are shown as mean + SEM (*n* = 6 Ctrl, 7 B-iKO). (**C** and **D**) Anti-dsDNA IgG ASC numbers in the spleen (**C**) and BM (**D**) analyzed by ELISPOT. (**E**) IgG and C3 deposits in the renal glomeruli analyzed by immunofluorescence microscopy. MFI of IgG and C3 was quantified. (**F**) PAS-stained glomerular sections and pathological scores. (**G**) Survival rates of Ctrl (*n* = 5) and B-iKO (*n* = 8) mice. (**H**) Transwell migration of PBs from RSQ-treated *Rosa26^CreERT2/+^Commd3^fl/+^* (Ctrl) and *Rosa26^CreERT2/+^Commd3^fl/fl^* (iKO) mice in response to the CXCR4 ligand. Data are shown as mean ± SD of technical triplicates. (**I**) Numbers of transferred anti-dsDNA IgG ASCs from RSQ-treated Ctrl and iKO mice that homed to the BM, analyzed by ELISPOT. Data are shown as percentage relative to the number of transferred ASCs and as a ratio relative to the number of transferred ASCs detected in the blood. (**J**) Frequency of ABCs in the kidneys of Ctrl and B-iKO BM chimeras described in Figure 6A, analyzed by flow cytometry. (**K**) Transwell migration of ABCs from RSQ-treated Ctrl and iKO mice in response to the CXCR3 ligand. Data are shown as mean ± SD of technical triplicates. (**L**) Numbers of transferred ABCs from RSQ-treated Ctrl and iKO mice that migrated to the kidney, analyzed by flow cytometry. Data are shown as percentage relative to the number of transferred ABCs. Data are shown as means, with symbols representing individual mice (**C**–**F**, **I**, **J**, and **L**). In **E**, gray symbols represent individual glomeruli. In **E** and **F**, representative images are shown (scale bar: 50 μm). Results are representative of 2 independent experiments. *P* values were determined by 2-way ANOVA with Šídák’s post hoc test (**B**, **H**, and **K**), 2-tailed unpaired *t* test (**C**–**F**, **I**, **J**, and **L**), or log-rank test (**G**). *P < 0.05; **P < 0.01; ***P < 0.001; ****P < 0.0001.

To assess the contribution of the COMMD3/8 complex expressed in B cells, we used the mixed BM chimeras generated with BM cells from μMT and *Rosa26^CreERT2/+^Commd3^fl/fl^* mice (Figure 6A). Tamoxifen administration after 4 weeks of RSQ treatment reduced autoantibody titers and autoreactive ASC numbers, ameliorated renal pathology, and prolonged survival (Figure 6, B–G). Notably, the magnitude of these improvements was comparable with that observed with systemic COMMD3/8-complex deficiency, underscoring an important B cell–intrinsic role for the COMMD3/8 complex in lupus pathology.

Since the COMMD3/8 complex plays a pivotal role in EF responses, we examined whether it also contributes to disease through GC responses. *S1pr2^CreERT2/+^Commd3^fl/fl^* mice were administered tamoxifen, beginning after 4 weeks of RSQ treatment, to induce GC B cell–specific deletion of *Commd3* (Supplemental Figure 5H). We confirmed efficient deletion of COMMD3 and COMMD8 at the protein level in GC B cells (Supplemental Figure 5I). GC B cell–specific COMMD3/8-complex deficiency reduced the number of GC B cells in the spleen (Supplemental Figure 5J), whereas the numbers of ABCs and PBs remained unchanged (Supplemental Figure 5, K and L), consistent with their predominantly GC-independent generation in this model. Importantly, autoantibody titers, autoreactive ASC numbers, and renal pathology were unaffected (Supplemental Figure 5, M–Q). These results suggest that the COMMD3/8 complex may contribute to lupus pathogenesis primarily through EF responses in this model, whereas its role in GC responses appears to be limited.

We then examined whether the COMMD3/8 complex is involved in the trafficking of pathogenic B cells to peripheral effector sites. CXCR4 directs autoreactive ASCs to the BM (36), where they can mature into long-lived PCs that sustain chronic autoantibody production (37, 38). COMMD3/8-complex deficiency impaired PB chemotaxis toward the CXCR4 ligand and reduced BM homing of autoreactive ASCs in the TLR7-induced lupus model (Figure 6, H and I).

Beyond lymphoid organs, ABCs also accumulate in the kidneys of patients with SLE, where their abundance correlates with nephritis severity (6, 39). ABCs upregulate inflammatory chemokine receptors such as CXCR3, facilitating renal infiltration (6). COMMD3/8-complex deficiency reduced ABC accumulation in the kidneys of RSQ-treated mice (Figure 6J). Consistently, COMMD3/8 complex–deficient ABCs exhibited impaired CXCR3-mediated chemotaxis and reduced trafficking to the kidney (Figure 6, K and L).

Thus, disruption of the COMMD3/8 complex ameliorates pathology in the TLR7-induced lupus model and is associated with impaired trafficking of pathogenic B cells, both within secondary lymphoid organs and toward peripheral effector sites such as the BM and kidney.

### Targeting the COMMD3/8 complex ameliorates lupus pathology in spontaneous models.

To extend our findings beyond the TLR7-driven model, we examined the impact of COMMD3/8-complex deficiency in *Fas^lpr^* mice on a C57BL/6 (B6) background (B6.*Fas^lpr^*) — a spontaneous lupus model in which autoantibodies are predominantly produced via EF responses with minimal GC responses (40, 41). Tamoxifen was administered to *Fas^lpr/lpr^Rosa26^CreERT2/+^Commd3^fl/fl^* mice at 20 weeks of age, when anti-dsDNA antibodies were detectable (Supplemental Figure 6A). COMMD3/8-complex deficiency reduced autoantibody titers and autoreactive ASC numbers, ameliorated glomerular injury, and improved survival (Supplemental Figure 6, B–G). Notably, IRF4^hi^Ki67^+^ ABCs were detected in the spleens of B6.*Fas^lpr^* mice, and their frequency was selectively reduced by COMMD3/8-complex deficiency, with a concomitant reduction in PBs (Supplemental Figure 6, H–J). These findings support the contribution of EF-derived pre-PB ABCs to lupus pathogenesis in murine models.

**Figure 7:**
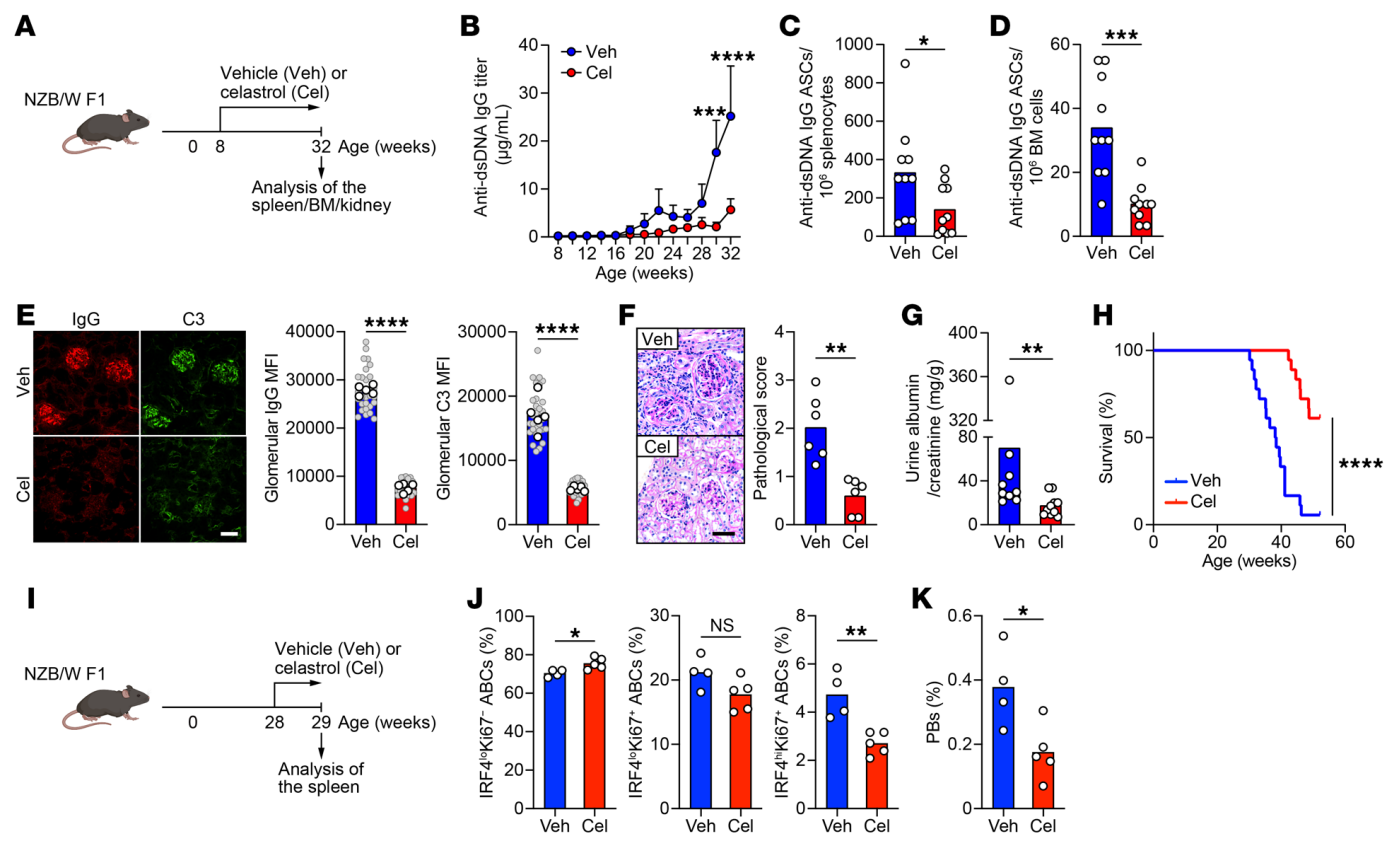
Effects of COMMD3/8 complex inhibition on spontaneous lupus. (**A**) Experimental diagram for **B**–**H**. NZB/W F1 mice were treated with vehicle (Veh) or celastrol (Cel). (**B**) Serum anti-dsDNA IgG titers analyzed by ELISA. Data are shown as mean + SEM (*n* = 10). (**C** and **D**) Anti-dsDNA IgG ASC numbers in the spleen (**C**) and BM (**D**) analyzed by ELISPOT. (**E**) IgG and C3 deposits in the renal glomeruli analyzed by immunofluorescence microscopy. MFI of IgG and C3 was quantified. (**F**) PAS-stained glomerular sections and pathological scores. (**G**) Urine albumin-to-creatinine ratio analyzed by ELISA. (**H**) Survival rates of Veh (*n* = 18) and Cel (*n* = 18) mice. (**I**) Experimental diagram for **J** and **K**. NZB/W F1 mice were treated daily with vehicle or celastrol, and splenocytes were analyzed by flow cytometry. (**J** and **K**) Frequencies of ABC populations in total ABCs (**J**) and of PBs in splenocytes (**K**). Data are shown as means, with symbols representing individual mice (**C**–**G**, **J**, and **K**). In **E**, gray symbols represent individual glomeruli. In **E** and **F**, representative images are shown (scale bar: 50 μm). Results are representative of 2 independent experiments. *P* values were determined by 2-way ANOVA with Šídák’s post hoc test (**B**), 2-tailed unpaired *t* test (**C**–**F**, **J**, and **K**), 2-tailed Mann–Whitney *U* test (**G**), or log-rank test (**H**). *P < 0.05; **P < 0.01; ***P < 0.001; ****P < 0.0001.

We next evaluated the therapeutic potential of pharmacologically targeting the COMMD3/8 complex using celastrol, a small molecule previously reported to inhibit the COMMD3/8 complex (17), in F1 hybrids of New Zealand Black × New Zealand White (NZB/W F1) mice. NZB/W F1 mice represent a polygenic lupus model that recapitulates key features of human SLE, including severe nephritis with proteinuria (42). Celastrol treatment initiated at 8 weeks of age suppressed autoantibody production, decreased autoreactive ASC numbers, attenuated glomerular injury and proteinuria, and improved survival (Figure 7, A–H). Importantly, celastrol selectively reduced IRF4^hi^Ki67^+^ ABCs and suppressed PB generation (Figure 7, I–K), recapitulating the effects of COMMD3/8-complex deficiency and suggesting that celastrol targets the COMMD3/8 complex–dependent EF differentiation program.

Taken together, these findings demonstrate that both genetic and pharmacologic inhibition of the COMMD3/8 complex ameliorates disease in multiple murine lupus models, suggesting that the COMMD3/8 complex may represent a promising therapeutic target in SLE.

## Discussion

In this study, we identified a transcriptionally and functionally distinct ABC state with PB precursor characteristics in both patients with SLE and murine lupus models. We show that EBI2-mediated ABC migration to EF niches within the splenic bridging channels provides a spatial cue promoting pre-PB ABC formation and autoreactive PB output. We further show that the COMMD3/8 complex is required not only for EBI2-dependent ABC positioning but also for multiple B cell migratory processes, including ASC homing to the BM and ABC infiltration into the kidney, supporting its role in lupus pathogenesis. Together, these findings uncover a spatially coordinated mechanism that drives the EF differentiation of ABCs into autoreactive PBs and contributes to the tissue distribution of pathogenic B cells, highlighting a pathway with therapeutic relevance in SLE (Supplemental Figure 7).

EF responses in lupus are increasingly recognized to be regulated at multiple levels. Previous work has established that TLR7 signaling, BCR engagement, and T cell–derived cytokines such as IFN-γ and IL-21 promote ABC generation (9, 10). A recent study further demonstrated that continuous CD21–complement engagement licenses naive Fo B cells to initiate ABC development and subsequently differentiate into ASCs (43). Moreover, the m^6^A demethylase fat mass and obesity-associated protein supports ABC expansion by enhancing mitochondrial oxidative metabolism (44). In concert with these pathways, the migration-dependent program described here likely represents an additional layer of regulation that promotes PB differentiation from ABCs and contributes to autoantibody production.

In patients with SLE, peripheral blood ABCs are enriched for a PC-associated gene signature compared with switched memory B cells (5). Extending these observations, our single-cell transcriptomic and BCR repertoire analyses revealed heterogeneity within the ABC compartment and identified a distinct pre-PB ABC state characterized by elevated expression of PC-associated genes, including *IRF4* and *MKI67*, as well as enrichment of autoreactive clones. Importantly, the frequency of this state correlated with autoantibody titers and disease activity, underscoring its clinical relevance in human SLE. These findings suggest that pre-PB ABCs may serve as a biomarker of autoreactive B cell activation associated with EF responses and as a potential indicator of therapeutic responsiveness.

Recapitulating the key features of human pre-PB ABCs, we identified an analogous cell state in the TLR7-induced lupus model, as well as in B6.*Fas^lpr^* and NZB/W F1 mice, suggesting that the pre-PB ABC state may be conserved across lupus models driven by distinct pathogenic mechanisms. A recent study in the MRL.*Fas^lpr^* lupus model described a proliferative population comprising both ABCs and PBs (26). Although pre-PB ABCs may be encompassed within this broader proliferative pool, our analysis defines them as a distinct cell state within the ABC compartment, transcriptionally distinguishable from PBs. Consistent with the differentiation pathway delineated in this study, prior work in a Lyn-deficient lupus model showed that IRF4 expression in T-bet–expressing B cells — a population that largely corresponds to ABCs — is required for autoreactive PC generation (45), supporting an IRF4-driven program that promotes PB differentiation from ABCs.

Our BCR repertoire analyses in patients with SLE and murine lupus models showed limited clonal overlap between ABC-3 and either ABC-1 or ABC-2. One possibility is that ABC-3 may arise from a different precursor population rather than from ABC-1 or ABC-2. Alternatively, this pattern may reflect clonal selection, in which a subset of autoreactive clones preferentially expands and progresses toward PB differentiation. This interpretation is consistent with a previous study demonstrating clonal continuity between ABCs and PBs, together with reduced clonal diversity (26). In this context, ABC-3 may represent a pre-PB state enriched in selected autoreactive clones, thereby exhibiting reduced clonal overlap with earlier ABC states that retain broader clonal diversity.

Comparing EF responses between species is complicated by differences in tissue sampling (murine spleen versus human peripheral blood) and by anatomical distinctions between murine and human spleens. In mice, the splenic bridging channels are thought to serve as EF niches enriched in EBI2 ligands, whereas functionally equivalent structures in humans remain less clearly defined (46). Nonetheless, recent studies suggest that the human spleen can support robust EF responses. Substantial ABC accumulation has been observed in the spleens of patients with autoimmune lymphoproliferative syndrome caused by *FAS* mutations (47), and ABCs localize to EF sites in severe COVID-19, a setting associated with heightened EF activity (48). Considering the high EBI2 expression and ligand responsiveness of human ABCs, analogous EF microenvironments may exist within the human spleen. High-resolution spatial transcriptomic approaches will be instrumental in identifying such sites and elucidating their contribution to EF responses in human SLE.

Our findings suggest that ABC migration to EF niches within the splenic bridging channels promotes pre-PB ABC generation. This process may, at least in part, be supported by interactions with cDC2s via TACI-mediated trophic signals. Previous studies showed in MRL.*Fas^lpr^* and NZB/W F1 lupus models that EF helper T cells accumulate in the bridging channels and promote PB differentiation through IL-21 and CD40 ligand (CD40L) (41). Similarly, in patients with SLE, peripheral helper T cells have been reported to participate in EF responses and potentially facilitate PB differentiation via IL-21 (49). These observations suggest that EF-localized helper T cells may also contribute to PB differentiation of ABCs. Therefore, impaired ABC positioning due to EBI2 or COMMD3/8-complex deficiency may hinder their interactions with the EF-resident T cells as well as cDC2s, attenuating PB output.

We note that the reduction in PBs observed in B cell–specific EBI2-deficient BM chimeras was greater than that observed following transfer of EBI2-deficient ABCs. The adoptive transfer experiments primarily assessed ABC-dependent PB differentiation, whereas the BM chimera experiments captured the entire process of B cell activation and differentiation. Therefore, the difference in PB reduction between the 2 experimental systems suggests that EBI2 may promote PB differentiation not only through its effect on ABC migration but also via additional mechanisms. Notably, previous studies suggest that a fraction of PBs may arise directly from activated B cells without transitioning through the ABC stage and localize to EF niches in an EBI2-dependent manner (21, 22). In this context, EBI2 deficiency may also impair ABC-independent PB differentiation, contributing to the more pronounced reduction in PBs in BM chimeras.

B cell–specific deficiency of the COMMD3/8 complex resulted in greater phenotypic improvement in the TLR7-induced lupus model than B cell–specific EBI2 deficiency. Beyond EBI2, the COMMD3/8 complex mediates signaling downstream of several chemoattractant receptors, including CCR7 and CXCR4 (16). Accordingly, its deficiency may affect CCR7-dependent migration of early activated B cells to the T-B boundary, potentially limiting their interactions with helper T cells and differentiation into pathogenic B cells. In this study, we showed that COMMD3/8-complex deficiency impaired CXCR4-mediated chemotaxis of ASCs and their homing to the BM. COMMD3/8-complex deficiency also reduced CXCR3-mediated chemotaxis of ABCs, accompanied by decreased renal infiltration. This broader role of the COMMD3/8 complex in B-lineage cell migration may account for the more pronounced effects of COMMD3/8-complex deficiency compared with EBI2 deficiency.

We previously found that disruption of the COMMD3/8 complex modestly reduced disease severity in collagen-induced arthritis (17). Notably, COMMD3/8-complex deficiency preferentially impairs the migration of B-lineage cells, although the molecular basis remains unclear (17). Because multiple immune and stromal cell types are involved in collagen-induced arthritis, the B cell–selective effect of COMMD3/8-complex deficiency may limit its overall impact. In contrast, SLE is predominantly driven by autoreactive B cells, providing a context in which B cell–focused interventions may exert more pronounced effects. Consistent with this, COMMD3/8-complex deficiency in the present study markedly suppressed PB responses and autoantibody production and was associated with reduced disease pathology in multiple lupus models. These findings suggest that targeting the COMMD3/8 complex may be particularly effective in B cell–driven autoimmune diseases.

COMMD3 and COMMD8 function as components of the Commander complex, which mediates the endocytic recycling of specific cell surface proteins (50–52). However, major chemoattractant receptors in B-lineage cells — CXCR4, CXCR5, CCR7, EBI2, and CXCR3 — lack the canonical sorting motifs required for Commander complex–mediated trafficking. Moreover, COMMD3/8 complex–deficient B cells exhibit normal surface expression and recycling of β1-integrin (17), a membrane protein whose recycling depends on the Commander complex. These observations suggest that COMMD3 and COMMD8 have a Commander complex–independent role in chemoattractant receptor signaling. We therefore speculate that COMMD3 and COMMD8 may also function in distinct molecular complexes from the canonical Commander complex.

Despite these insights, our study has several limitations. First, although pre-PB ABCs correlated with clinical parameters, the cohort included relatively few patients who were treatment naive and individuals with high disease activity. In addition, a subset of individuals deviated from these trends, suggesting that disease pathogenesis may not be fully explained by this population alone. Second, it was technically challenging to reliably identify pre-PB ABCs in situ in the spleens of RSQ-treated mice. We therefore employed adoptive transfer approaches. However, the transfer procedure itself might have altered ABC localization. Third, although most pre-PB ABCs were generated in a GC-independent manner, a contribution of GC-derived pre-PB ABCs and GC responses to disease pathogenesis cannot be formally excluded. Finally, we utilized TLR7-induced and *Fas^lpr^* lupus models on a B6 background, which recapitulate key features of EF responses. However, these models exhibit relatively mild kidney pathology and do not fully capture the severity and heterogeneity of SLE. Thus, caution is warranted when extrapolating these findings to human disease.

Overall, our study defines a migration-dependent checkpoint in the EF differentiation of autoreactive B cells and suggests that targeting this program may offer therapeutic opportunities in antibody-mediated autoimmune diseases.

## Methods

### Sex as a biological variable.

Our study examined male and female human participants and mice. Sex was not considered a primary biological variable, and no sex-stratified analyses were performed.

### Human samples.

Patients with SLE who met the 2019 European League Against Rheumatism/American College of Rheumatology classification criteria (53) were recruited from Kobe University Hospital. Clinical characteristics of the study participants are summarized in Supplemental Table 1 (scRNA-seq) and Supplemental Table 2 (flow cytometry). Patient samples were analyzed as they became available, without prespecified exclusion criteria based on clinical characteristics.

### Mice.

B6 mice were purchased from CLEA Japan. B6.*Fas^lpr^* mice and NZB/W F1 mice were purchased from Japan SLC. CD45.1^+^, *Rosa26^loxP-STOP-loxP-tdTomato/+^*, and μMT (129S2-*Ighm^tm1Cgn^*) mice on the B6 background were obtained from The Jackson Laboratory. Blimp1-mVenus mice on the B6 background [B6.Cg-Tg(Prdm1-Venus)] (Acc. No. CDB0460T) (54) were obtained from RIKEN BRC. *Irf4^GFP^* mice on the B6 background (55) were provided by A. Kallies (The University of Melbourne). *Rosa26^CreERT2/+^* mice on the B6 background [*Gt(ROSA)26Sor^tm9(Cre/ESR1)Arte^*] were obtained from Taconic Biosciences. *S1pr2^CreERT2/+^* mice (25) were bred with *Rosa26^loxP-STOP-loxP-tdTomato^* mice to produce *S1pr2*-tdTomato mice. *Gpr183^fl/fl^* mice on the B6 background (56) were provided by T. Willinger (Karolinska Institute) and bred with *Rosa26^CreERT2/+^* mice to produce *Rosa26^CreERT2/+^Gpr183^fl/fl^* mice. *Commd3^fl/fl^* mice were generated as previously described (16) and bred with *Rosa26^CreERT2/+^* and *S1pr2^CreERT2/+^* mice to produce *Rosa26^CreERT2/+^Commd3^fl/fl^* and *S1pr2^CreERT2/+^Commd3^fl/fl^* mice, respectively. *Fas^lpr/lpr^Rosa26^CreERT2/+^Commd3^fl/fl^* mice were generated by crossing B6.*Fas^lpr^* mice with *Rosa26^CreERT2/+^Commd3^fl/fl^* mice. Mice were 8–12 weeks old at the start of experiments unless otherwise indicated. Experiments were performed using sex-matched littermates, and both sexes were included across experiments. Mice were housed in a specific pathogen-free facility.

### Plasmids.

Sense (S) and antisense (AS) oligonucleotides encoding the following sgRNAs (coding sequences underlined) were cloned into the pSpCas9(BB)-2A-GFP plasmid (PX458; Addgene, 48138) containing the sgRNA scaffold backbone, Cas9, and GFP: sg*COMMD3*_S, 5′-CAC CGAC TCT GGA ATG CCG CCC GG-3′; sg*COMMD3*_AS, 5′-AAA CCCG GGC GGC ATT CCA GAG TC-3′. Construct integrity was verified by Sanger sequencing.

### Antibodies.

An antibody against COMMD3 (HPA036584) was purchased from Merck. Antibodies against COMMD8 (N-15, sc-104159) and GAPDH (FL-335, sc-25778) were purchased from Santa Cruz Biotechnology. The following purified, fluorophore-conjugated, or biotin-conjugated antibodies (BioLegend) were used for flow cytometry, cell sorting, and immunofluorescence microscopy: anti-mouse CD3-biotin (17A2, 100244); anti-mouse CD4-biotin (GK1.5, 100404); anti-mouse CD8α-biotin or -FITC (53-6.7, 100704 or 100706); anti-mouse CD11b-FITC (M1/70, 101206); anti-mouse CD11c-allophycocyanin (APC) or -phycoerythrin/cyanine7 (PE/Cy7) (N418, 117310 or 117318); anti-mouse purified CD16/32 (93, 101302); anti-mouse purified CD16/32 (TruStain FcX PLUS; S17011E, 156604); anti-mouse CD19-PE or -Brilliant Violet (BV) 421 (6D5, 152408 or 115549); anti-mouse CD23-FITC or -PE/Cy7 (B3B4, 101606 or 101614); anti-mouse CD38-PE/Cy7 (90, 102717); anti-mouse CD45R/B220-FITC, APC/Cy7, or -BV510 (RA3-6B2; 103206, 103224, or 103248); anti-mouse CD45.1-biotin or -Alexa Fluor (AF) 647 (A20, 110704 or 110720); anti-mouse CD45.2-biotin, PE, or -BV421 (104; 109804, 109808, or 109832); anti-mouse CD93-APC (AA4.1, 136510); anti-mouse CD138-PE, -APC, -BV605, or -BV421 (281-2; 142504, 142505, 142516, or 142523); anti-mouse CD169-AF488 (3D6.112, 142419); anti-mouse CD268 (BAFFR)-AF647 (7H22-E16, 134105); anti-mouse CXCR3-APC or -PE/Cy7 (CXCR3-173, 126512 or 126515); anti-mouse GL7-FITC or -AF647 (GL7, 144604 or 144606); anti-mouse IgD-AF647 (11-26c.a, 405707); anti-mouse IgG-AF647 (Poly4053, 405322); anti-mouse IgG2b-FITC (RMG2b-1, 406706); anti-mouse/human IRF4-PE or -AF647 (IRF4.3E4, 646403 or 646408); anti-mouse I-A/I-E (MHC-II)-FITC, APC/Cy7, or -BV510 (M5/114.15.2; 107606, 107627, or 107635); anti-mouse Ki67-AF647, -AF488, or -PE/Cy7 (16A8, 652407, 652417, or 652426); anti-mouse Ly6G-biotin (1A8, 127604); anti-mouse 33D1 (DCIR2)-biotin (33D1, 124903); anti-human CD11c-PE/Cy7 (S-HCL-3, 371507); anti-human purified CD16/32/64 (TruStain FcX; 3G8/FUN-2/10.1, 422302); anti-human CD19-FITC (HIB19, 302256); anti-human CD21-PE (Bu32, 354903); anti-human CD24-APC/Cy7 (ML5, 311132); anti-human CD27-PE or -PE/Cy7 (O323, 302807 or 302837); anti-human CD38-APC or -BV785 (HIT2, 303509 or 303529); anti-human GPR183 (EBI2)-APC (SA313E4, 368907); anti-human IgD-BV421 or -BV605 (IA6-2, 348226 or 348231); and anti-human Ki67-AF488 (Ki-67, 350507). The following antibodies were purchased from BD Biosciences: anti-mouse CD3ε-BV605 (145-2C11, 563004); anti-mouse CD11b-BV605 or -Brilliant Ultraviolet (BUV) 395 (M1/70, 563015 or 565976); anti-mouse CD11c-BV605 or -BUV737 (N418, 744179 or 749039); anti-mouse CD19-BV786 or -BUV737 (1D3, 563333 or 612781); anti-mouse CD21/CD35-BV605 or -BUV737 (7G6, 563176 or 612810); anti-mouse CD23-BUV395 (B3B4, 740216); anti-mouse CD45R/B220-BUV395 (RA3-6B2, 563793); anti-mouse CD45.2-BUV737 (104, 612778); anti-mouse CD95-PE/Cy7 or -BUV737 (Jo2, 557653 or 741763); anti-mouse CD267 (TACI)-PE or -BV421 (8F10, 558410 or 742840); anti-mouse CD287 (TLR7)-PE (A94B10, 565557); anti-mouse IgD-BV605 (11-26c.2a, 563003); anti-mouse IgG1-FITC (A85-1, 553443); anti-mouse IgG3-FITC (R40-82, 553403); anti-mouse active caspase-3-AF647 (C92-605, 560626); anti-human CD11c-BV421 (B-ly6, 562561); anti-human CD19-BUV395 or -BV786 (HIB19, 740287 or 740968); and anti-human CD21-BUV737 (B-ly4, 612789). An antibody against mouse IgG2c-FITC (STAR135F) was purchased from Bio-Rad Laboratories. An antibody against mouse complement C3-PE (RmC11H9, CL7503PE) was purchased from Cedarlane. An antibody against mouse EBI2 (A-20, sc-66436) was purchased from Santa Cruz Biotechnology. An antibody against rabbit IgG (H+L)-biotin (111-065-144) was purchased from Jackson ImmunoResearch Laboratories. The following oligo-tagged antibodies (BioLegend) were used for cell hashing in scRNA-seq experiments: anti-mouse TotalSeq-C0301 Hashtag 1 (M1/42, 30-F11; 155861); anti-mouse TotalSeq-C0302 Hashtag 2 (M1/42, 30-F11; 155863); anti-mouse TotalSeq-C0303 Hashtag 3 (M1/42, 30-F11; 155865); anti-mouse TotalSeq-C0304 Hashtag 4 (M1/42, 30-F11; 155867); anti-human TotalSeq-C0251 Hashtag 1 (LNH-94, 2M2; 394661); and anti-human TotalSeq-C0252 Hashtag 2 (LNH-94, 2M2; 394663).

### Flow cytometry and cell sorting.

Spleens and BM were disrupted by passing through 40-μm and 70-μm cell strainers, respectively (Corning, 352340 and 352350), and treated with ammonium chloride potassium buffer to lyse RBCs. Single-cell suspensions were blocked with anti-Fc receptor antibodies and stained with fluorophore- or biotin-conjugated antibodies. For intracellular staining of IRF4, Ki67, and active caspase-3, surface-stained cells were fixed with the Fixation Buffer (BD Biosciences, 554655) for 30 minutes at 4°C and permeabilized with the Fixation and Permeabilization Buffer (BD Biosciences, 554714) for 30 minutes at 4°C, followed by incubation with antibodies against the respective intracellular antigens. Non-viable cells were labeled using 7-amino-actinomycin D (7-AAD; BioLegend, 420403), Zombie Aqua Fixable Viability Kit (BioLegend, 423101), or Zombie NIR Fixable Viability Kit (BioLegend, 423105). Flow cytometry data were acquired using the LSRFortessa X-20 (BD Biosciences) and analyzed with FlowJo software (v10.10.0, BD Biosciences). Cell sorting was performed using the FACSAria II, FACSAria Fusion, or FACSMelody (all from BD Biosciences).

### Immunofluorescence microscopy.

Spleens were fixed in 4% paraformaldehyde (Thermo Fisher Scientific, 43368) in PBS (FUJIFILM Wako, 166-23555) for 2 hours at 4°C. Frozen sections (10 μm thick) of fixed spleens and unfixed kidneys were blocked with 0.1% globulin-free BSA (FUJIFILM Wako, 013-15104) in PBS and stained for B220, CD3ε, CD45.2, CD138, CD169, IgD, IRF4, and DCIR2 in the spleens, and for C3 and IgG in the kidneys. For DCIR2 staining, tyramide signal amplification was performed using the Biotin XX Tyramide SuperBoost Kit (Thermo Fisher Scientific, B40931) according to the manufacturer’s instructions. Sections were mounted with the FluorSave Reagent (Merck, 34578920ML). Images were acquired at room temperature using the FLUOVIEW FV3000 inverted confocal microscope (EVIDENT) equipped with FV31S-SW software (v2.5.1.228) with the Plan Apochromat UPLXAPO objective lens (20 × with 0.80 NA or 40 × with 0.95 NA for spleen sections; 40 × with 0.95 NA for kidney sections). Immunofluorescence intensity was quantified using Fiji software (v2.16.0/1.54p) by investigators blinded to genotype and treatment.

### Statistics.

Statistical analyses were performed using GraphPad Prism 8 or 9 (GraphPad Software) except for scRNA-seq data analyses. Statistical parameters, including the exact *n* value (with the description of what *n* represents), the mean, median, and measures of dispersion (SD or SEM), are reported in the figures and figure legends. Unless otherwise stated, statistical significance was determined by the 2-tailed paired or unpaired Student’s *t* test, 2-tailed Mann–Whitney *U* test, 1-way ANOVA with Tukey’s or Dunnett’s post hoc test, or 2-way ANOVA with Šídák’s post hoc test. Correlations between 2 variables were assessed using the 2-tailed Pearson’s correlation test. Survival curves were compared using the log-rank test. *P* and *P_adj_* values less than 0.05 were considered statistically significant.

### Study approval.

All procedures involving human samples were approved by the Institutional Review Boards of Kobe University Hospital (approval no. B240091) and the WPI Immunology Frontier Research Center, The University of Osaka (approval no. 2020-16). Written informed consent was obtained from all participants prior to enrollment after they received a thorough explanation of the study. All mouse experiments were performed in accordance with protocols approved by the Animal Care and Use Committee of the WPI Immunology Frontier Research Center, The University of Osaka (approval no. R7-07-0).

### Data availability.

Raw scRNA/BCR-seq data have been deposited in the Gene Expression Omnibus under accession number GSE307415. This study does not report any original code. All other data are available in the main text or the supplemental materials. Values for all data points in graphs are reported in the Supporting Data Values file.

## Author contributions

TS, KK, and KS conceived the study. TS and KK designed and performed the experiments, analyzed the data, and wrote the manuscript. The order of co-first authorship was determined based on overall contributions to the study. MK developed the human B cell culture system and conducted the related experiments. SI collected and curated samples and clinical information from patients with SLE. SS provided the dsDNA antigen and anti-dsDNA antibody used in ELISA. AN provided the sgRNAs used for plasmid construction. SL contributed to the analysis of scRNA/BCR-seq data and edited the manuscript. YCL, DM, and DO performed scRNA/BCR-seq. MN and AK contributed to the analysis of patient-derived data. TK provided mice and reagents. JS contributed to experiments involving patient samples. KS supervised the study and wrote the manuscript.

## Conflict of interest

The authors have declared that no conflict of interest exists.

## Funding support

- Japan Agency for Medical Research and Development (JP18gm6210007, JP20ak0101129, JP20ek0410072, JP24ym0126815, and JP25gm4010029 to KS).
- Japan Society for the Promotion of Science, Grants-in-Aid for Scientific Research (JP19H03489 and JP22H00451 to KS; JP19K16690, JP22K07118, and JP25K00293 to AN; JP24K18465 to TS).
- Japan Science and Technology Agency, Moonshot R&D Program (JPMJMS2023 to KS).
- Takeda Science Foundation (to KS and AN).
- Chemo-Sero-Therapeutic Research Institute (to KS).
- Terumo Life Science Foundation (to KS).
- KANAE Foundation for the Promotion of Medical Science (to AN).
- Japanese Society for Immunology, KIBOU project fellowship (to TS).

## Supplementary Material

Supplemental data

Unedited blot and gel images

Supporting data values

## Version 1 07/14/2026

In-Press Preview

## Version 2 08/17/2026

Electronic publication
