# Supplementary material for: Migration-dependent extrafollicular programming of preplasmablast age-associated B cells drives lupus pathogenesis
Source: J Clin Invest. 2026 Jul 14;136(16):e204095. doi: 10.1172/JCI204095 (PMC13476141; doi:10.1172/JCI204095)
Supplement: Supplementary file 1 [file jci-136-204095-s038.pdf]

Supplemental methods

Isolation of human PBMCs

PBMCs were isolated from the heparinized whole blood of SLE patients and healthy donors without a history of autoimmune disease. Briefly, the blood samples were diluted two-fold with PBS containing 2% FBS (Thermo Fisher Scientific, 10270-106) and layered over a lymphocyte separation solution (Nacalai Tesque, 20828-15) in SepMate tubes (VERITAS, ST-86450). Following centrifugation at 1200 × g for 10 minutes at room temperature, the PBMC layer was collected and washed twice with PBS containing 2% FBS.

Generation of BM chimeras

BM chimeras were generated by irradiating B6 mice with a single dose of 8 Gy, followed by intravenous transfer of 4×10^6 BM cells from μMT mice together with 1×10^6 BM cells from either *Rosa26*$^{CreERT2/+}$*Gpr183*$^{flox/+}$ or *Rosa26*$^{CreERT2/+}$*Gpr183*$^{flox/flox}$ mice, or from either *Rosa26*$^{CreERT2/+}$*Commd3*$^{flox/+}$ or *Rosa26*$^{CreERT2/+}$*Commd3*$^{flox/flox}$ mice. Chimeras were analyzed 8–12 weeks after BM reconstitution.

Tamoxifen-induced gene deletion

Deletion of *Commd3* in *Rosa26*$^{CreERT2/+}$*Commd3*$^{flox/flox}$ and *Fas*$^{lpr/lpr}$*Rosa26*$^{CreERT2/+}$*Commd3*$^{flox/flox}$ mice, as well as deletion of *Gpr183* in *Rosa26*$^{CreERT2/+}$*Gpr183*$^{flox/flox}$ mice, was induced by oral administration of 2 mg tamoxifen (Merck, T5648) dissolved in sunflower oil (Merck, S5007) for 3 consecutive days. For long-term gene deletion, the 3-day regimen of tamoxifen treatment was repeated every 12 weeks. To induce GC B cell-specific expression of Cre recombinase, *S1pr2*$^{CreERT2/+}$*Commd3*$^{flox/flox}$ (for *Commd3* deletion) and *S1pr2*-tdTomato mice (for tdTomato expression) were administered tamoxifen every 2 days (25).

RSQ treatment

Mice were treated epicutaneously with 20 μg RSQ (Merck, SML0196) in 20 μl acetone on the left ear three times a week at 1- or 2-day intervals.

Celastrol treatment

Mice received intraperitoneal injections of vehicle (PBS containing 2% DMSO) or celastrol (Merck, C0869; 1 mg/kg, twice a week at 2- and 3-day intervals) unless otherwise indicated.

dsDNA bait preparation

dsDNA bait was prepared as previously described (57). Briefly, a 500-bp DNA fragment was amplified from mouse genomic DNA using KOD-Plus-Neo polymerase (TOYOBO, KOD-401) with a 5'-biotinylated forward primer (5'-Biotin-GGACCCAACCCAGGAGGCAGATGT-3') and an unmodified reverse primer (5'-CCTCTAAGGCTTCGCTGTTATTACCAC-3'). The PCR product was purified by agarose gel electrophoresis followed by gel extraction, and its concentration was measured using the NanoDrop

spectrophotometer (Thermo Fisher Scientific). As a control bait, HEL (Merck, L6876) was biotinylated using the EZ-Link Sulfo-NHS-Biotinylation Kit (Thermo Fisher Scientific, 21425) according to the manufacturer's instructions. Each biotinylated bait was diluted to 250 nM in PBS and incubated with PE- or BV421-conjugated streptavidin (BioLegend, 405204 or 405225) at a 5:1 molar ratio for 30 minutes at 4 °C. Splenocytes were stained with the fluorochrome-conjugated bait at a final concentration of 30 nM.

EdU labeling and detection

In vivo labeling with 5-ethynyl-2'-deoxyuridine (EdU) was performed as previously described (58). Mice were intravenously injected with 1 mg EdU 45 minutes prior to sacrifice. Splenocytes were labeled for incorporated EdU using the Click-iT EdU Alexa Fluor 488 Flow Cytometry Assay Kit (Thermo Fisher Scientific, C10425) according to the manufacturer's instructions, and analyzed by flow cytometry.

UMAP visualization of flow cytometry data

Flow cytometry data were pre-gated on live $CD19^+$ cells. UMAP dimensionality reduction was performed on compensated fluorescence parameters using the UMAP plugin (v4.1.1) available from the FlowJo Exchange (BD Biosciences). Cell populations corresponding to individual UMAP clusters were identified by manual gating and overlaid onto the UMAP plot.

scRNA/BCR-seq

Sample preparation and cell hashing

For the human ABC/PB sample in Figure 1, ABCs and PBs were sorted from PBMCs of a patient with SLE. Each cell population was labeled with a distinct cell-hashing antibody—TotalSeq-C0251 and -C0252, respectively—according to the manufacturer's instructions, and pooled at a 4:1 ratio. For the mouse FoB/GCB/ABC/PB sample described in Figure 2, Fo B cells, GC B cells, ABCs, and PBs were sorted from the spleens of two WT mice treated with RSQ for 4 weeks. Each cell population was labeled with TotalSeq-C0301, -C0302, -C0303, and -C0304, respectively, and pooled in equal numbers. For the mouse ABC sample in Supplemental Figure 4, the mixed BM chimeras were administered tamoxifen on days 28–30 of RSQ treatment, and spleens were harvested on day 35. ABCs were sorted from the spleen of one mouse per genotype, labeled with TotalSeq-C0301 and -C0303, respectively, and pooled in equal numbers.

Library construction and sequencing

Single-cell suspensions were processed using the Chromium Controller (10x Genomics) according to the Chromium Next GEM Single Cell 5' Reagent Kits with Feature Barcoding technology for Cell Surface Protein and Immune Receptor Mapping User Guide. Reagents (all from 10x Genomics) included the Chromium Next GEM Single Cell 5' Kit v2 (1000263), Chromium 5' Feature Barcode Kit (1000541), Chromium Next GEM Chip K Single Cell Kit (1000287), Dual Index Kit TT Set A (1000215), and Dual Index Kit TN Set A (1000250). Briefly, single-cell gel bead-in-emulsions were generated using the

Chromium Controller, and cDNA was synthesized via reverse transcription in the Veriti Thermal Cycler (Thermo Fisher Scientific). Resulting cDNA, including that derived from mRNA and hashtag oligonucleotide (HTOs), was tagged with cell-specific barcodes and unique molecular identifiers. Libraries for gene expression and feature barcodes were amplified and quantified using the High Sensitivity DNA Kit (Agilent Bioanalyzer, 5067-4626). Amplified cDNA was enzymatically fragmented, end-repaired, and polyadenylated, followed by cleanup and size selection using SPRIselect magnetic beads (Beckman Coulter, B23317). The final libraries were constructed after ligation of sequencing adapters (Illumina), index amplification, and double-sided size selection with SPRIselect magnetic beads. For BCR repertoire profiling, full-length VDJ segments were enriched using constant region-targeting primers with the Chromium Single Cell Mouse or Human BCR Amplification Kit (10x Genomics, 1000255 or 1000253). Libraries were quality-checked with the High Sensitivity DNA Kit and sequenced on the NovaSeq 6000 or NovaSeq X Plus (Illumina) using paired-end reads.

Analysis of scRNA/BCR-seq data

Preprocessing

Raw sequencing data were processed with Cell Ranger (10x Genomics): v7.1.0 for the mouse FoB/GCB/ABC/PB dataset, and v9.0.1 for the mouse ABC and human ABC/PB datasets. FASTQ files were generated using the cellranger mkfastq function. Gene expression and VDJ libraries were processed using the cellranger count function, aligning reads to the following reference genomes: mm10-2020-A/vdj_GRCm38_alts_ensembl-7.0.0 for the mouse FoB/GCB/ABC/PB dataset, GRCm39-2024-A for the mouse ABC dataset, and GRCh38-2024-A/vdj_GRCh38_alts_ensembl-7.1.0 for the human ABC/PB dataset.

Cell clustering and gene expression analysis

Downstream analysis was performed using the Seurat package (v4.3.0) (59) in R (v4.2.2). Cells expressing fewer than 200 genes or with > 5% mitochondrial reads were filtered out. HTO demultiplexing was performed using the HTODemux function after centered log-ratio normalization via the NormalizeData function. Cells were classified as singlets, doublets, or negative, and only singlets were retained. For cell-cycle scoring, the CellCycleScoring function was used to assign phase-specific scores. The G2M–S difference was regressed out during scaling via the vars.to.regress parameter in the ScaleData function. Data were normalized using the LogNormalize method, and 2000 highly variable genes were selected with the FindVariableFeatures function using the vst method. Principal component analysis (PCA) was performed on scaled data using the RunPCA function. UMAP dimensionality reduction and clustering were conducted using the RunUMAP, FindNeighbors, and FindClusters functions with the top 20 principal components. Clustering resolution was set to 0.5 for the mouse FoB/GCB/ABC/PB dataset, 0.1 for the mouse ABC dataset, and 0.3 for the human ABC/PB dataset. UMAP visualizations were generated with the DimPlot function. Cluster-specific marker genes were identified using the FindAllMarkers function (Wilcoxon rank-sum test, Bonferroni correction). Statistically

significant DEGs were defined as those with an average log2 fold change > 0.25, P_{adj} < 0.05, and expressed in > 25% of cluster cells. Cell types were annotated based on gene expression and hashing, and similar clusters were merged. Violin and dot plots were generated using the VlnPlot and DotPlot functions, respectively. Heatmaps were created using the DoHeatmap function, displaying the top 10 DEGs per cluster ranked by average log2 fold change.

Pathway enrichment analysis

For mABC-1, mABC-2, and mABC-3 clusters, DEGs were identified with the FindAllMarkers function, applying the same significance thresholds as above. Pathway enrichment was performed with the compareCluster function in clusterProfiler (v4.6.2) (60), using the following parameters: fun = enrichPathway (Reactome), pvalueCutoff = 0.05, and pAdjustMethod = BH.

GSEA

GSEA was conducted using pre-ranked gene lists generated by the FindMarkers function (Wilcoxon rank-sum test). Genes expressed in > 1% of cells were ranked by average log2 fold change. GSEA (v4.4.0, Broad Institute) was run using the following gene sets: GSE13411_PLASMA_CELL_VS_MEMORY_BCELL_UP, KEGG_APOPTOSIS, WP_TOLLLIKE_RECEPTOR_SIGNALING, and REACTOME_SIGNALING_BY_THE_B_CELL_RECEPTOR_BCR from MSigDB, as well as the custom gene set IRF4_TARGETS (5). NES and nominal *P* value were reported.

BCR repertoire analysis

BCR repertoire analysis was performed using the Immcantation framework (v4.5.0) (61). VDJ gene segments were assigned using IgBLAST via Change-O (v1.3.0) (61) against the IMGT reference. Nonproductive sequences and cells with multiple heavy chains or only light chains were excluded. Data were integrated with Seurat objects, and cells lacking cell-type assignment were removed. Clones with IgG isotypes were retained. Mouse data were analyzed with scRepertoire (v2.0.0) (62) using the clonalOverlap function and a “strict” clone definition (identical VDJ gene usage and complementarity determining region 3 nucleotide sequence). Human data were analyzed with Immunarch (v1.0.0) using the repOverlap function and a “v” clone definition (identical V gene usage). IGHV4-34-expressing clones were quantified with the countGenes function in Alakazam (v1.2.1) (61).

ELISA

For measurement of serum anti-dsDNA IgG titers, dsDNA antigen was prepared as follows. Calf thymus DNA (Merck, D3664-5X2MG) was sonicated into fragments < 3 kbp in length and treated with 1 U/μg S1 nuclease (Takara Bio, 2410A) for 30 minutes at 37°C. The resulting mixture was treated with Tris-EDTA-saturated phenol (Nippon Gene, 313-90091) and subjected to ethanol precipitation. MaxiSorp plates (Thermo Fisher Scientific, 439454) were coated with 5 μg/ml dsDNA antigen overnight at 4°C and

blocked with 0.5% globulin-free BSA in PBS containing 0.05% Tween-20 (PBS-T). Serially diluted serum was plated in duplicate and incubated for 2 hours at room temperature. After washing with PBS-T, wells were incubated with an HRP-conjugated anti-mouse IgG antibody (Southern Biotech, 1030-05) for 1 hour at room temperature. The HRP activity was detected with a 3,3',5,5'-tetramethylbenzidine substrate (Seracare Life Sciences, 5120-0077) and absorbance at 450 nm was measured using the Multiskan FC microplate photometer (Thermo Fisher Scientific) after quenching the reaction with 1 N HCl. The high-affinity anti-dsDNA antibody (121G9) (63) was used to generate a standard curve for quantification. Urinary albumin and creatinine levels were measured using the Albwell M (Ethos Biosciences, 1011) and Creatinine Companion (Ethos Biosciences, 1012) kits, respectively.

ELISPOT

MultiScreen HTS HA plates (Merck, 20-168100110) were coated with 10 μg/ml methylated BSA (Merck, A1009-1G) for 2 hours at 37°C under 5% CO_2, followed by 5 μg/ml dsDNA antigen (prepared as described in the ELISA section) overnight at 4°C. Serially diluted spleen or BM cells (0.0625–1 × 10^6 cells/well) were plated in duplicate and incubated for 16 hours at 37°C under 5% CO_2 in RPMI 1640 medium (FUJIFILM Wako, 189-02025) supplemented with 10% FBS and 55 μM 2-ME. ABCs sorted from the spleens of RSQ-treated *Irf4*^{GFP} mice (4 weeks of treatment) were serially diluted (0.25–0.5 × 10^5 cells/well), plated in duplicate, and incubated for 24 hours in the same supplemented medium containing 1 μg/ml RSQ. After washing with PBS-T, wells were incubated with a goat anti-mouse IgG antibody (Southern Biotech, 1030-01) in PBS-T containing 1% Blocking One (Nacalai Tesque, 03953-66) for 2 hours at room temperature, followed by an alkaline phosphatase-conjugated anti-goat IgG antibody (Southern Biotech, 6160-04) for 2 hours at room temperature. Spots were developed using a 5-bromo-4-chloro-3-indolyl-phosphate/nitro blue tetrazolium (Promega, S3771) and enumerated by investigators blinded to genotype and treatment.

Histopathology

Kidneys were fixed in 4% paraformaldehyde in PBS overnight at 4°C. Paraffin-embedded sections (4 μm thick) were stained with PAS reagent and evaluated by light microscopy as previously described (64). Glomerular pathology was assessed by investigators blinded to genotype and treatment, with 20 glomeruli examined per kidney, and the mean score was calculated. Each glomerulus was scored as follows: 0, normal (35–40 cells); 1, mild (mild hypercellularity, 41–50 cells); 2, moderate (moderate hypercellularity, 51–60 cells, including segmental and/or diffuse proliferative changes); 3, severe (severe hypercellularity, > 60 cells, including segmental or global sclerosis and/or crescent formation). Images were acquired using NIS-Elements BR software (v4.30.00) with the CFI Plan APO Lambda objective lens (40 × with 0.95 NA) on the H550L light microscope (Nikon).

Human B cell culture and electroporation

Human B cells were isolated from the PBMCs of healthy donors by negative selection using the B Cell Isolation Kit II (Miltenyi Biotec, 130-091-151) on the autoMACS Pro Separator (Miltenyi Biotec). Cells were cultured at 1 × 10^6 cells/ml in 24-well plates (Thermo Fisher Scientific, 142475) at 37°C under 5% CO_2 in Iscove's modified Dulbecco's medium (FUJIFILM Wako, 098-06465) supplemented with 10% FBS, 55 μM 2-ME, MEGACD40L (100 ng/ml; Enzo Life Sciences, ALX-522-110), recombinant human IL-2 (50 ng/ml; BioLegend, 589104), IL-10 (50 ng/ml; Thermo Fisher Scientific, 200-10), IL-15 (10 ng/ml; Thermo Fisher Scientific, 200-15), and CpG oligodeoxynucleotide 2006 (1 μg/ml; InvivoGen, tlrl-2006), as previously described (65). Forty-eight hours later, cells were washed with PBS and resuspended at 2 × 10^7 cells/ml in the Neon Buffer R (Neon NxT Electroporation System 10-μl Kit; Thermo Fisher Scientific, N1025) containing 5 μg of sgRNA-cloned pSpCas9(BB)-2A-GFP plasmid per 1 × 10^6 cells. Electroporation was performed using the Neon NxT Transfection System (Thermo Fisher Scientific, NEON1S) with three 10-ms pulses at 1550 V. Immediately after electroporation, cells were cultured at 1 × 10^6 cells/ml in RPMI 1640 medium supplemented with 10% FBS, 55 μM 2-ME, 2 mM L-glutamine (FUJIFILM Wako, 073-05391), 2 mM sodium pyruvate (Nacalai Tesque, 06977-34), and 2 mM HEPES (Dojindo, 345-06681), along with an ABC-skewing cocktail containing RSQ (1 μg/ml), recombinant human CD40L (10 μg/ml; BioLegend, 591706), BAFF (20 ng/ml; BioLegend, 559602), IL-2 (10 ng/ml; BioLegend, 589102), IL-21 (10 ng/ml; BioLegend, 571202), IFN-γ (20 ng/ml; BioLegend, 570204), and anti-human IgA + IgG + IgM $F(ab')_2$ fragment (10 μg/ml; Jackson ImmunoResearch Laboratories, 109-006-064) (11). Forty-eight hours after electroporation, $DAPI^-GFP^+$ cells were sorted using the FACSMelody and cultured for an additional 48 hours in the same medium with the ABC-skewing cocktail. The cells were then harvested for immunoblotting, flow cytometry, or migration assays.

Immunoblot

To assess the levels of COMMD3, COMMD8, and GAPDH, GFP^+ cells or GC B cells were lysed in lysis buffer consisting of 1% NP-40 (Nacalai Tesque, 23640-94), 150 mM NaCl, 10 mM Tris-HCl (pH 7.2), and a protease inhibitor cocktail (Nacalai Tesque, 25955-11). Reduced samples were resolved by SDS-PAGE using NuPAGE Bis-Tris gels (Thermo Fisher Scientific, NP0336) and transferred to Immobilon-P membranes (Merck, ISEQ00010). Membranes were blocked with 1% globulin-free BSA in TBS containing 0.1% Tween-20 (TBS-T) and blotted with primary antibodies, followed by an HRP-conjugated anti-rabbit or anti-goat IgG antibody (Jackson ImmunoResearch Laboratories, 711-035-152 or 805-035-180). Signals were developed with ECL substrate (Cytiva, RPN2232) and visualized with the Amersham Imager 800 luminescence image analyzer (Cytiva). Band densities were quantified using Fiji software (v2.16.0/1.54p) and normalized to GAPDH.

Transwell migration assay

Spleens were harvested from WT mice after 4 weeks of RSQ treatment for migration assays in Figure 3C. To assess the effects of COMMD3/8 complex deficiency, $Rosa26^{CreERT2/+}Commd3^{flox/+}$ and $Rosa26^{CreERT2/+}Commd3^{flox/flox}$ mice were administered tamoxifen on days 28–30 of RSQ treatment.

Spleens were collected on day 32 for migration assays in Figure 6H and on day 35 for migration assays in Figures 5A and 6K. Splenocytes were treated with ammonium chloride potassium buffer to lyse RBCs, washed five times, and incubated for 30 minutes at 37°C in RPMI 1640 medium containing 0.5% fatty acid-free BSA (Merck, 126575). Human PBMCs, isolated as described above, were processed similarly except without RBC lysis. Cells were seeded into the upper chambers of 24-well or 96-well transwells (Corning, 3421 or 3387) and allowed to migrate for 3 hours at 37°C across 5-μm pore filters toward the following chemoattractants placed in the lower chambers: 7α,25-HC (Merck, SML0541), 7α,27-HC (Avanti, 700024P), CXCL12 (R&D Systems, 460-SD), or CXCL9 (R&D Systems, 492-MM). Cells that migrated to the lower chambers were enumerated by flow cytometry and chemotactic responses were calculated as percentages relative to the input cell numbers. In Figure 3, C and E, responses of different cell types were normalized by subtracting the number of cells that migrated in the absence of chemoattractants from the total number of migrated cells.

In vitro differentiation of B cells

Rosa26$^{CreERT2/+}$*Commd3*$^{flox/+}$ and *Rosa26*$^{CreERT2/+}$*Commd3*$^{flox/flox}$ mice were administered tamoxifen on days 0–2, and spleens were harvested on day 7. B cells were isolated from splenocytes by negative selection using CD43 microbeads (Miltenyi Biotec, 130-049-801) on the autoMACS Pro Separator. Purified B cells were cultured at 5 × 10^6 cells/ml in 96-well round-bottom plates (Corning, 3799) for 48 hours at 37°C under 5% CO_2 in RPMI 1640 medium supplemented with 10% FBS, 55 μM 2-ME, and 2 mM L-glutamine, and stimulated with 1 μg/ml RSQ, 5 μg/ml anti-mouse IgM $F(ab')_2$ fragment (Jackson ImmunoResearch Laboratories, 115-006-020), and 100 ng/ml recombinant mouse IFN-γ (Thermo Fisher Scientific, 315-05). The frequencies of ABCs and PBs were analyzed by flow cytometry.

Antigen presentation assay

Rosa26$^{CreERT2/+}$*Commd3*$^{flox/+}$ and *Rosa26*$^{CreERT2/+}$*Commd3*$^{flox/flox}$ mice were administered tamoxifen on days 28–30 of RSQ treatment, and spleens were harvested on day 35. Splenocytes were pulsed with 10 μg/ml MHC-II Eα chain (52–68) peptide (Eα peptide; ASFEAQGALANIAVDKA; AnaSpec, AS-61621) at 37 °C for 1 hour, as previously described (66). Cells were labeled with a biotin-conjugated antibody against the Eα peptide:I-A^b complex (Y-Ae; Thermo Fisher Scientific, 13-5741-81) followed by streptavidin-BV421 (BioLegend, 405226) and analyzed by flow cytometry.

Coculture of cDC2s with ABCs

Spleens were harvested from WT mice after 4 weeks of RSQ treatment. ABCs were sorted from the splenocytes using the FACSAria II or FACSAria Fusion. For the isolation of cDC2s ($CD3^-CD19^-Ly6G^-CD11c^{hi}MHC\text{-}II^{hi}CD8^-CD11b^+$), spleens were minced and digested in RPMI 1640 medium supplemented with 2% FBS, 0.25 mg/ml collagenase type 4 (Worthington Biochemical, CLS4), and 20 μg/ml DNase I (Roche, 10104159001) for 10 minutes at 37°C in a water bath. Digestion was quenched with 2 mM EDTA, and the resulting cell suspensions were passed through a 70-μm cell strainer. Cells were labeled

with biotin-conjugated antibodies against CD3, CD19, and Ly6G, followed by incubation with streptavidin microbeads (Miltenyi Biotec, 130-048-101), and subjected to negative selection on the autoMACS Pro Separator. cDC2s were sorted from the negatively selected fraction using the FACSAria II or FACSAria Fusion. For the coculture assay, ABCs (0.5×10^5 cells/well) were seeded into the lower chambers and cDC2s (1×10^5 cells/well) into the upper chambers of 96-well transwell plates with 0.4-μm pore inserts (Corning, 3381). Cells were cultured at 37°C under 5% CO_2 in RPMI 1640 medium supplemented with 10% FBS, 55 μM 2-ME, and 2 mM L-glutamine, and stimulated with 0.5 μg/ml RSQ. After 24 hours of coculture, ABCs were harvested and analyzed by flow cytometry. To assess the role of TACI-mediated signaling, recombinant mouse TACI-Fc chimera protein (30 μg/ml; R&D Systems, 1041-TC) was added to the coculture system.

Adoptive transfer of ABCs

Splenocytes were collected from *Rosa26$^{CreERT2/+}$Commd3$^{flox/+}$* and *Rosa26$^{CreERT2/+}$Commd3$^{flox/flox}$* mice, or from *Rosa26$^{CreERT2/+}$Gpr183$^{flox/+}$* and *Rosa26$^{CreERT2/+}$Gpr183$^{flox/flox}$* mice, after 4 weeks of RSQ treatment. Cells were labeled with biotin-conjugated antibodies against CD4, CD8α, and Ly6G, followed by incubation with streptavidin microbeads, and subjected to negative selection on the autoMACS Pro Separator. ABCs were sorted from the negatively selected fraction using the FACSAria II or FACSAria Fusion and intravenously transferred (2×10^6 cells/mouse) into CD45.1$^+$ recipients that had been treated with RSQ for 1 week. Recipient mice were administered tamoxifen for 3 consecutive days, beginning the day after cell transfer. Spleens were harvested 14 days post-transfer and analyzed by immunofluorescence microscopy and flow cytometry. For flow cytometry, transferred cells were enriched by negative selection with a biotin-conjugated antibody against CD45.1 and streptavidin microbeads on the autoMACS Pro Separator. For experiments described in Figure 2, K and L, splenocytes from *Irf4^{GFP}* mice treated with RSQ for 4 weeks were processed similarly. GFP^{lo} or GFP^{hi} ABCs (1×10^6 cells/mouse) were intravenously transferred into CD45.1$^+$ recipients that had been treated with RSQ for 1 week, and the spleens were harvested 3 days later for flow cytometry. For experiments described in Figure 3F and Supplemental Figure 3B, splenocytes from WT mice treated with RSQ for 4 weeks were processed similarly. ABCs (2×10^6 cells/mouse) were intravenously transferred into untreated CD45.1$^+$ recipients, and the spleens were harvested 24 hours later for immunofluorescence microscopy.

BM homing of autoreactive ASCs

Rosa26$^{CreERT2/+}$Commd3$^{flox/+}$ and *Rosa26$^{CreERT2/+}$Commd3$^{flox/flox}$* mice were administered tamoxifen for 3 consecutive days after 8 weeks of RSQ treatment, and spleens were harvested one day after the final tamoxifen dose. Splenocytes were labeled with biotin-conjugated antibodies against CD4, CD8α, and Ly6G, followed by incubation with streptavidin microbeads, and subjected to negative selection on the autoMACS Pro Separator. The negatively selected fraction ($1.5–2.0 \times 10^8$ cells/mouse) was intravenously transferred into WT recipients. Twenty-four hours after cell transfer, blood and BM cells were collected

from the recipient mice. The numbers of anti-dsDNA IgG ASCs in the transferred cells, blood, and BM cells were measured by ELISPOT.

ABC migration to the kidney

Rosa26$^{CreERT2/+}$*Commd3*$^{flox/+}$ and *Rosa26*$^{CreERT2/+}$*Commd3*$^{flox/flox}$ mice were administered tamoxifen for 3 consecutive days after 8 weeks of RSQ treatment. Spleens were harvested one day after the final tamoxifen dose, and splenocytes (2–3 × 10^8 cells) were intravenously transferred into CD45.1$^+$ recipients that had been treated with RSQ for 1 week. Twenty-four hours after transfer, kidneys were collected from the recipient mice. For kidney dissociation, tissues were minced and placed into gentleMACS C Tubes (Miltenyi Biotec, 130-096-334) containing the enzyme mixture from the Multi Tissue Dissociation Kit 2 (Miltenyi Biotec, 130-110-201). Dissociation was performed using the gentleMACS Octo Dissociator with Heaters (Miltenyi Biotec), running the 37C_m_Kidney program. The resulting cell suspensions were filtered through a 100-μm MACS SmartStrainer (Miltenyi Biotec, 130-110-917), resuspended in Debris Removal Solution (Miltenyi Biotec, 130-109-398), and overlaid with 0.5% BSA and 2 mM EDTA in PBS. Lymphocytes were enriched from the bottom layer by density-gradient centrifugation at 2500 × g for 10 minutes, followed by two washes with PBS containing 2% FBS and 1 mM EDTA. The numbers of ABCs in the transferred cells and in the kidneys were measured by flow cytometry.

Supplemental references

57. Mayer CT, et al. An apoptosis-dependent checkpoint for autoimmunity in memory B and plasma cells. *Proc Natl Acad Sci USA.* 2020;117(40):24957–24963.
58. Liu D, et al. CD97 promotes spleen dendritic cell homeostasis through the mechanosensing of red blood cells. *Science.* 2022;375(6581):eabi5965.
59. Hao Y, et al. Integrated analysis of multimodal single-cell data. *Cell.* 2021;184(13):3573–3587.e29.
60. Wu T, et al. clusterProfiler 4.0: A universal enrichment tool for interpreting omics data. *Innovation (Camb).* 2021;2(3):100141.
61. Gupta NT, et al. Change-O: a toolkit for analyzing large-scale B cell immunoglobulin repertoire sequencing data. *Bioinformatics.* 2015;31(20):3356–3358.
62. Borcherding N, et al. scRepertoire: An R-based toolkit for single-cell immune receptor analysis. *F1000Res.* 2020;9:47.
63. Sakakibara S, et al. Clonal evolution and antigen recognition of anti-nuclear antibodies in acute systemic lupus erythematosus. *Sci Rep.* 2017;7(1):16428.
64. Kikawada E, et al. IL-12 deficiency in MRL-Fas^{lpr} mice delays nephritis and intrarenal IFN-γ expression, and diminishes systemic pathology. *J Immunol.* 2003;170(7):3915–3925.
65. Moffett HF, et al. B cells engineered to express pathogen-specific antibodies protect against infection. *Sci Immunol.* 2019;4(35):eaax0644.
66. Yin Y, et al. Jmjd1c demethylates STAT3 to restrain plasma cell differentiation and rheumatoid arthritis. *Nat Immunol.* 2022;23(9):1342–1354.

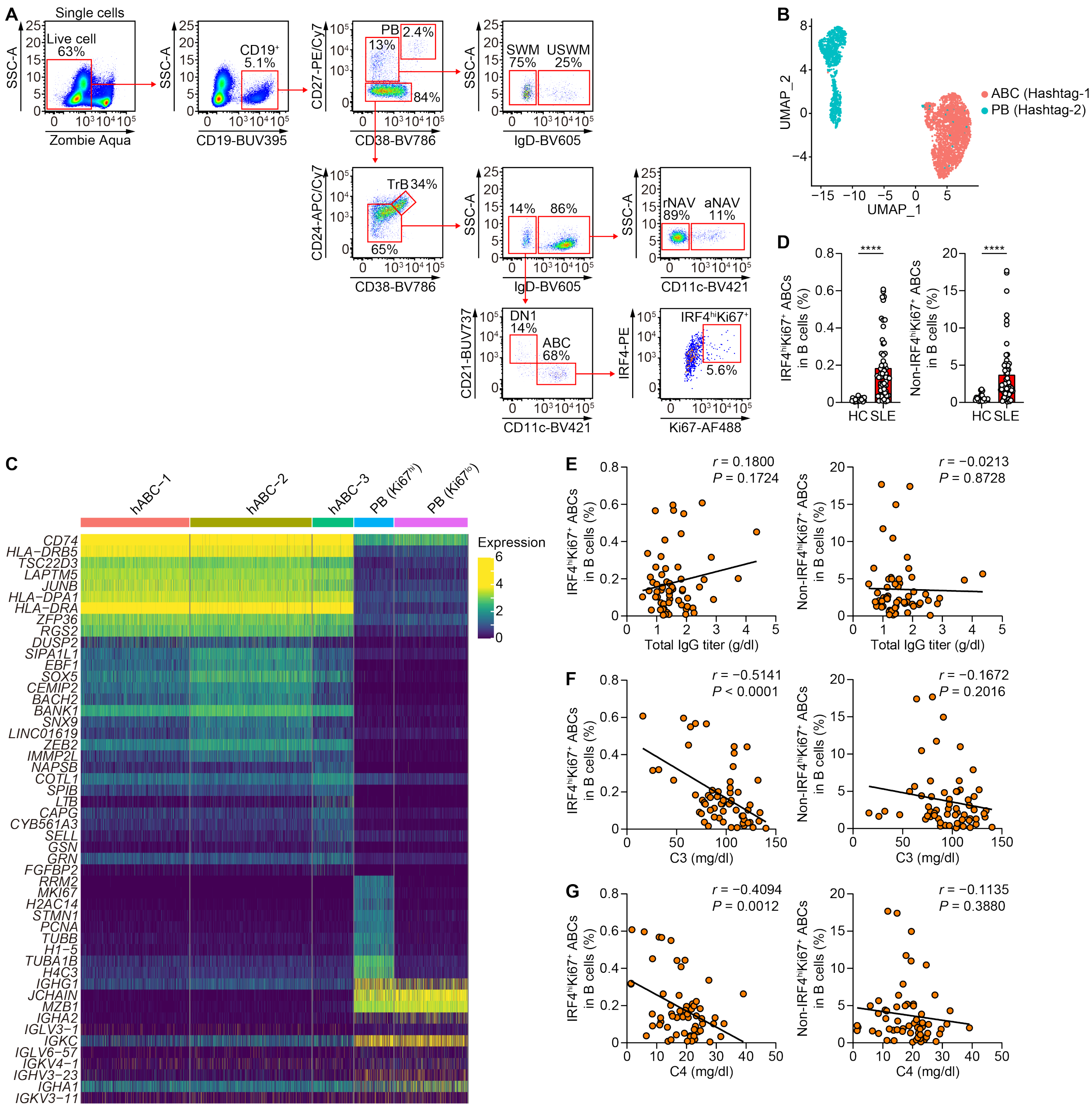

Supplemental Figure 1. Characterization of pre-PB ABCs in SLE patients. (**A**) Gating strategies for the indicated B-lineage populations in PBMCs from SLE patients. (**B** and **C**) scRNA-seq data related to Figure 1, A–F. (B) UMAP visualization colored by hashtags. (C) Heatmap showing normalized expression levels of the top 10 differentially expressed genes (DEGs) in each cluster. (**D**) Frequencies of IRF4^{hi}Ki67^{+} ABCs (left) and non-IRF4^{hi}Ki67^{+} ABCs (right) among B cells. Data are shown as means, with symbols representing individual HC or patients. (**E**–**G**) Correlations of IRF4^{hi}Ki67^{+} ABC frequency (left) or non-IRF4^{hi}Ki67^{+} ABC frequency (right) with serum total IgG titers (E), C3 levels (F), and C4 levels (G). Each point represents an individual patient, and the solid line indicates the linear regression fit. The correlation

coefficient *r* values were determined by two-tailed Pearson's correlation test. *P* values were determined by two-tailed Mann–Whitney *U* test (D) or two-tailed Pearson's correlation test (E–G). ****$P < 0.0001$.

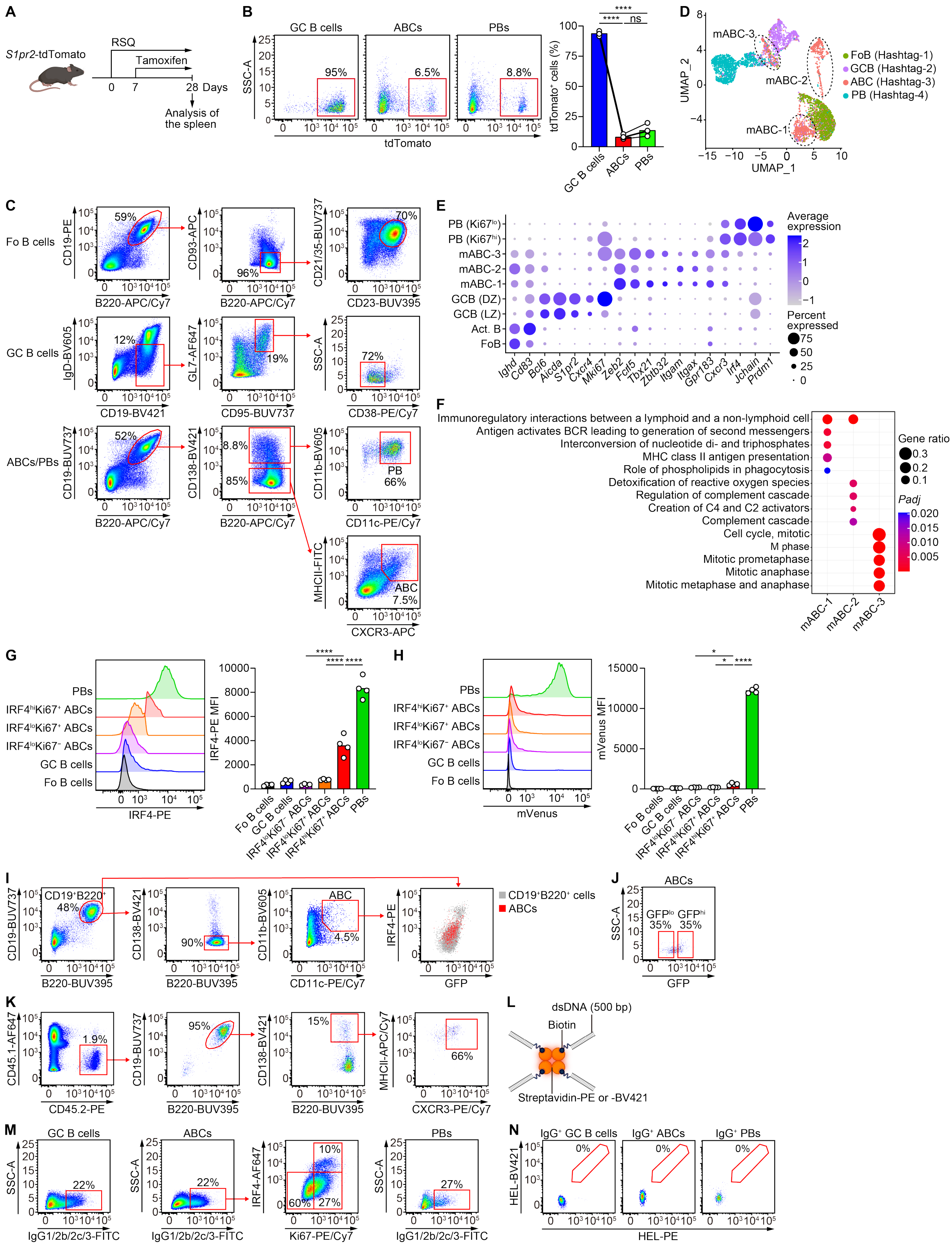
A
S1pr2-tdTomato
RSQ
Tamoxifen
0 7 28 Days
Analysis of the spleen
B
GC B cells
ABCs
PBs
SSC-A
95%
6.5%
8.8%
tdTomato
tdTomato+ cells (%)
****
****
ns
GC B cells
ABCs
PBs
C
Fo B cells
CD19-PE
59%
B220-APC/Cy7
CD93-APC
96%
CD21/35-BUV737
70%
CD23-BUV395
GC B cells
IgD-BV605
12%
CD19-BV421
GL7-AF647
19%
CD95-BUV737
72%
CD38-PE/Cy7
ABCs/PBs
CD19-BUV737
52%
CD138-BV421
8.8%
85%
CD11b-BV605
PB
66%
CD11c-PE/Cy7
MHCII-FITC
ABC
7.5%
CXCR3-APC
D
mABC-3
mABC-2
mABC-1
FoB (Hashtag-1)
GCB (Hashtag-2)
ABC (Hashtag-3)
PB (Hashtag-4)
UMAP_1
UMAP_2
E
PB (Ki67lo)
PB (Ki67hi)
mABC-3
mABC-2
mABC-1
GCB (DZ)
GCB (LZ)
Act. B
FoB
Ighd Cd83 Bcl6 Aicda S1pr2 Cxcr4 Mki67 Zeb2 Fcrl5 Tbx21 Zbtb32 Itgam Itgax Gpr183 Cxcr3 Irf4 Jchain Prdm1
Average expression
Percent expressed
F
Immunoregulatory interactions between a lymphoid and a non-lymphoid cell
Antigen activates BCR leading to generation of second messengers
Interconversion of nucleotide di- and triphosphates
MHC class II antigen presentation
Role of phospholipids in phagocytosis
Detoxification of reactive oxygen species
Regulation of complement cascade
Creation of C4 and C2 activators
Complement cascade
Cell cycle, mitotic
M phase
Mitotic prometaphase
Mitotic anaphase
Mitotic metaphase and anaphase
mABC-1
mABC-2
mABC-3
Gene ratio
Padj
G
PBs
IRF4hiKi67+ ABCs
IRF4loKi67+ ABCs
IRF4loKi67- ABCs
GC B cells
Fo B cells
IRF4-PE
IRF4-PE MFI
H
mVenus
mVenus MFI
I
CD19+B220+
48%
CD19-BUV737
B220-BUV395
CD138-BV421
90%
CD11b-BV605
ABC
4.5%
CD11c-PE/Cy7
IRF4-PE
GFP
CD19+B220+ cells
ABCs
J
ABCs
GFPlo 35%
GFPhi 35%
SSC-A
GFP
K
CD45.1-AF647
1.9%
CD45.2-PE
CD19-BUV737
95%
B220-BUV395
CD138-BV421
15%
MHCII-APC/Cy7
66%
CXCR3-PE/Cy7
L
dsDNA (500 bp)
Biotin
Streptavidin-PE or -BV421
M
GC B cells
22%
IgG1/2b/2c/3-FITC
ABCs
22%
IRF4-AF647
10%
60%
27%
Ki67-PE/Cy7
PBs
27%
N
IgG+ GC B cells
IgG+ ABCs
IgG+ PBs
0%
HEL-BV421
HEL-PE

Supplemental Figure 2. Characterization of pre-PB ABCs in TLR7-induced lupus. (**A** and **B**) *S1pr2*-tdTomato mice were administered tamoxifen from day 7 of RSQ treatment, and splenocytes were analyzed by flow cytometry on day 28 (A). Flow cytometry plots and frequencies of $tdTomato^+$ cells in the indicated B-lineage subsets are shown (B). (**C**) Gating strategies for B-lineage populations from the spleens of WT mice on day 28 of RSQ treatment. (**D**–**F**) scRNA-seq data related to Figure 2, A–G. (D) UMAP visualization colored by hashtags, with clusters of ABCs outlined. (E) Dot plots showing the expression of marker genes used for cell-type identification. (F) The top five enriched pathways for each cell type based on DEGs relative to other cell types. P_{adj}, adjusted *P*. (**G** and **H**) Flow cytometry of IRF4 (G) and mVenus (H) expression in the indicated B-lineage populations from the spleens of WT and Blimp1-mVenus mice, respectively, on day 28 of RSQ treatment. Representative histograms and MFI are shown. (**I**) Flow cytometry plots showing IRF4 and GFP expression in ABCs from the spleens of $Irf4^{GFP}$ mice on day 28 of RSQ treatment. (**J**) Gating strategy for GFP^{lo} and GFP^{hi} ABCs. (**K**) Gating strategy for identifying donor-derived ($CD45.2^+$) PBs in Figure 2L. (**L**) Schematic representation of the dsDNA bait. (**M**) Gating strategies for $IgG1^+$ cells in the indicated B-lineage populations from the spleens of WT mice on day 28 of RSQ treatment. (**N**) Flow cytometry plots showing hen egg lysozyme (HEL)-binding cells among IgG^+ B-lineage cells. HEL was used as a control antigen. Data are shown as means, with symbols representing individual mice (B, G, and H). Results are representative of two independent experiments. *P* values were determined by one-way ANOVA with Tukey's post hoc test. P_{adj} values were determined by Benjamini–Hochberg method. *$P < 0.05$; ****$P < 0.0001$; ns, not significant.

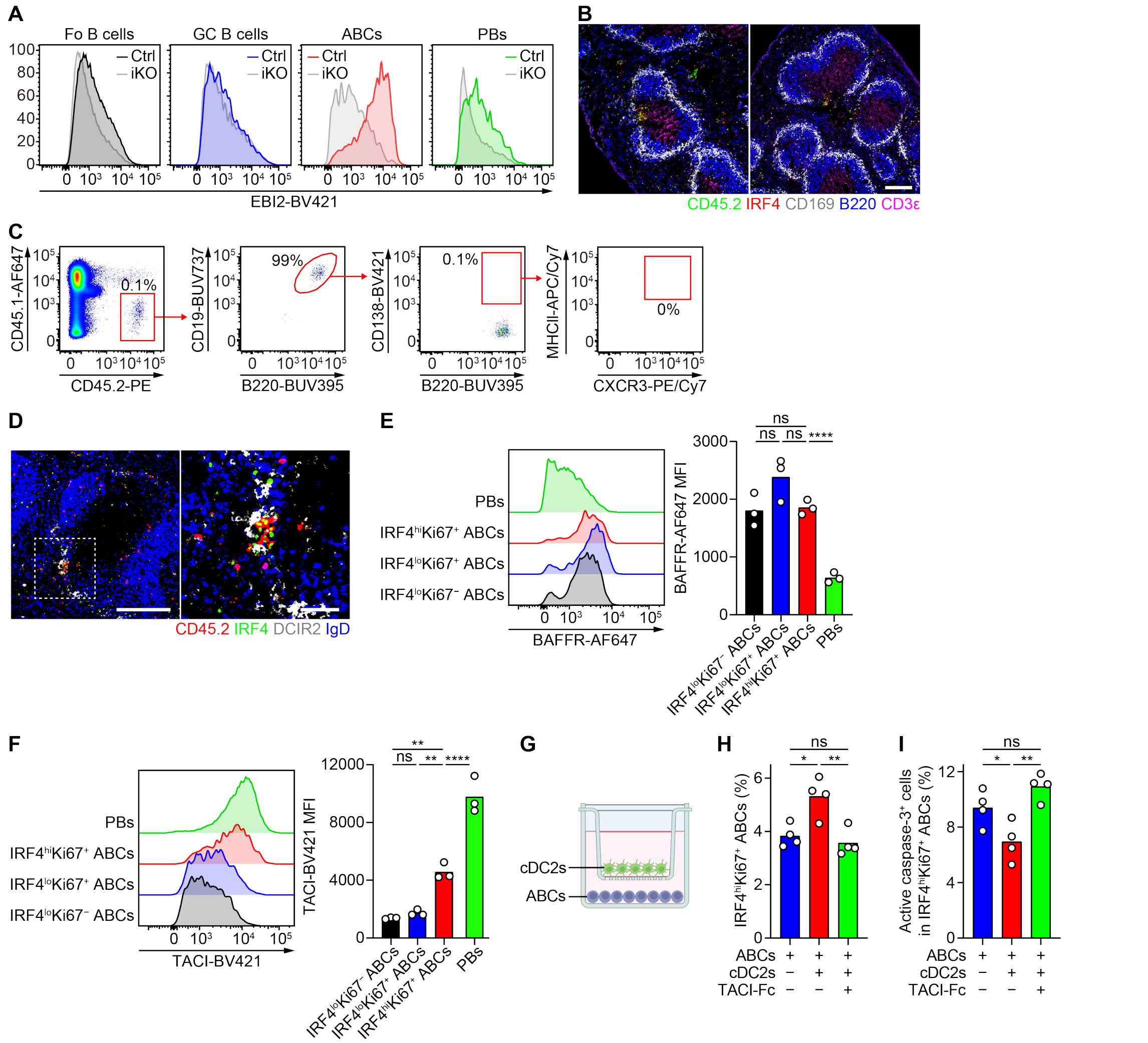

Supplemental Figure 3. Interaction between ABCs and cDC2s. (**A**) Validation of the anti-mouse EBI2 antibody. *Rosa26*$^{CreERT2/+}$*Gpr183*$^{+/+}$ (Ctrl) or *Rosa26*$^{CreERT2/+}$*Gpr183*$^{flox/flox}$ (iKO) mice were administered tamoxifen on days 28–30 of RSQ treatment, and splenic B-lineage cells were analyzed by flow cytometry on day 35. The staining pattern of GC B cells, which are known to lack EBI2 expression, was comparable between EBI2-sufficient and -deficient cells. In contrast, ABCs from Ctrl mice exhibited a clear shift in staining intensity compared with those from iKO mice. (**B**) Additional representative images from the experiment shown in Figure 3F (scale bar, 200 μm). (**C**) CD45.2$^+$ ABCs from RSQ-treated WT mice were transferred into CD45.1$^+$ WT recipients. Flow cytometry of CD45.2$^+$ cells in recipient spleens confirmed that transferred ABCs had not yet differentiated into PBs. (**D**) Splenic distribution of CD45.2$^+$ ABCs was analyzed similarly to Figure 3F, in relation to cDC2s (DCIR$^+$). Representative images are shown (left; scale bar, 200 μm), with boxed regions displayed at higher magnification (right; scale bar, 50 μm). (**E** and **F**) Flow cytometry of BAFF receptor (BAFFR) (E) and TACI (F) expression in ABC populations and PBs from the spleens of WT mice on day 28 of RSQ treatment. Representative histograms and MFI are shown. (**G**) Schematic of the non-contact coculture system of ABCs with cDC2s. (**H** and **I**) Frequencies of

$IRF4^{hi}Ki67^+$ ABCs in total ABCs (H) and of active caspase-3^+ cells in $IRF4^{hi}Ki67^+$ ABCs (I) under the indicated conditions. Data are shown as means, with symbols representing individual mice (E, F, H, and I). Results are representative of two independent experiments. *P* values were determined by one-way ANOVA with Tukey's post hoc test. **P* < 0.05; ***P* < 0.01; *****P* < 0.0001; ns, not significant.

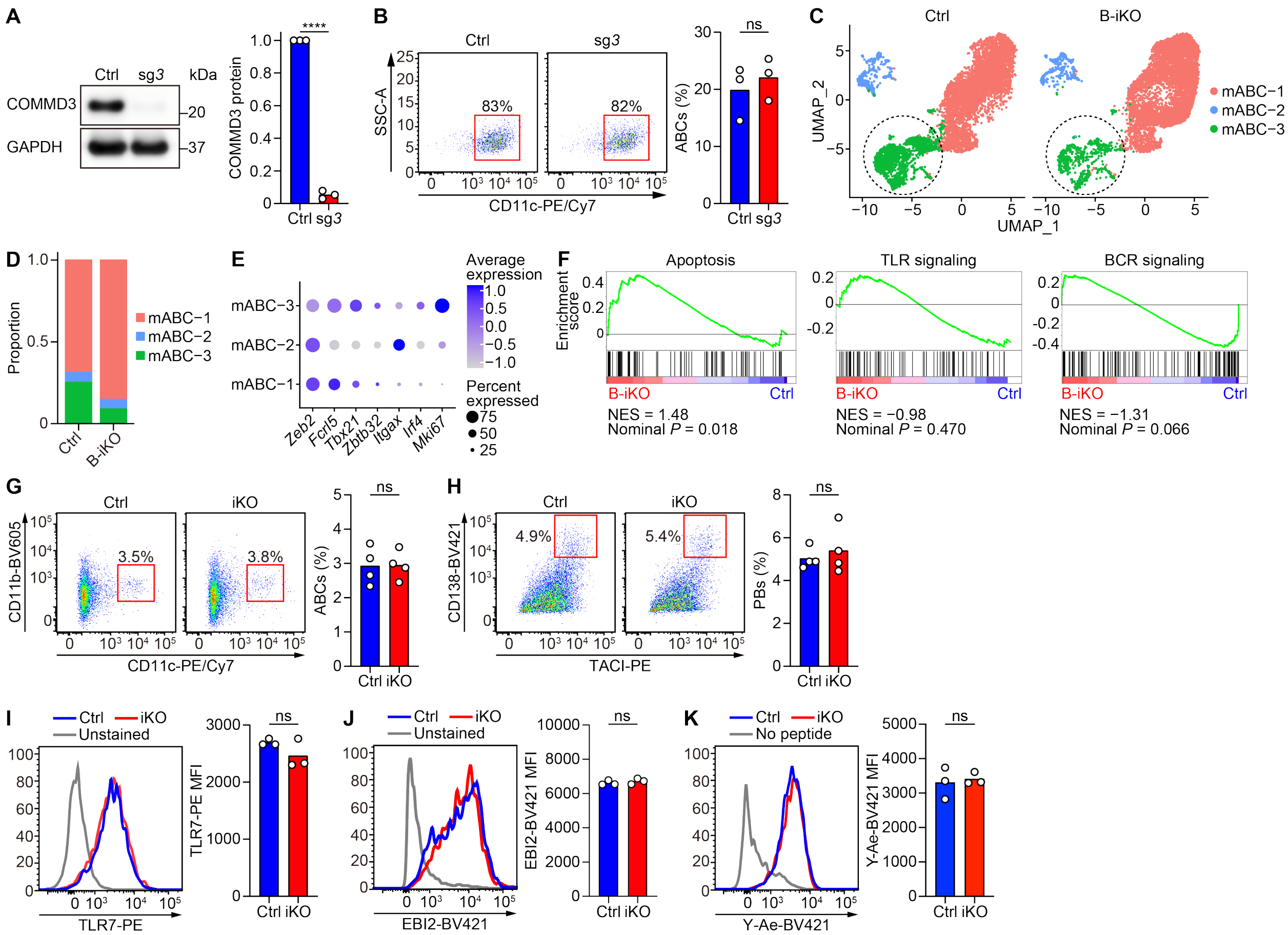

Supplemental Figure 4. Role of the COMMD3/8 complex in ABC functions. (**A**) Immunoblot analysis of COMMD3 protein levels in cells used in Figure 5C. Representative blots and quantification normalized to GAPDH are shown. (**B**) Frequency of ABCs in cells used in Figure 5C. Flow cytometry plots and quantification are shown. (**C**–**F**) Ctrl and B-iKO BM chimeras generated as in Figure 5G were administered tamoxifen on days 28–30 of RSQ treatment. ABCs were sorted from the spleens and subjected to scRNA-seq on day 35. (C and D) UMAP visualization colored by cell type, with mABC-3 circled (C) and the proportion of each cell type (D). (E) Dot plots showing the expression of marker genes used for cell-type identification. (F) GSEA comparing mABC-3 from Ctrl and B-iKO mice using the gene sets “APOPTOSIS” (left), “TOLLLIKE_RECEPTOR_SIGNALING” (middle), and “SIGNALING_BY_THE_B_CELL_RECEPTOR” (right). Data were obtained from 1 Ctrl and 1 B-iKO mouse. (**G**–**K**) *Rosa26*$^{CreERT2/+}$*Commd3*$^{flox/+}$ (Ctrl) and *Rosa26*$^{CreERT2/+}$*Commd3*$^{flox/flox}$ (iKO) mice were administered tamoxifen on days 28–30 of RSQ treatment, and spleens were harvested on day 35. (G and H) Splenic B cells were isolated and stimulated with RSQ, anti-IgM F(ab’)$_2$, and IFN-γ for 48 hours. Frequencies of ABCs (G) and PBs (H) were analyzed by flow cytometry. Flow cytometry plots and quantification are shown. (I and J) Expression of TLR7 (I) and EBI2 (J) in ABCs analyzed by flow cytometry. (K) Splenocytes were cultured with or without the Eα peptide, and the levels of the Eα peptide:MHC-II complex on ABCs were analyzed by flow cytometry using the Y-Ae antibody.

Representative histograms and MFI are shown (I–K). Data are shown as means, with symbols representing individual donors (A and B) or mice (G–K). Results are representative of two independent experiments. *P* values were determined by two-tailed unpaired *t* test. ****P < 0.0001; ns, not significant.

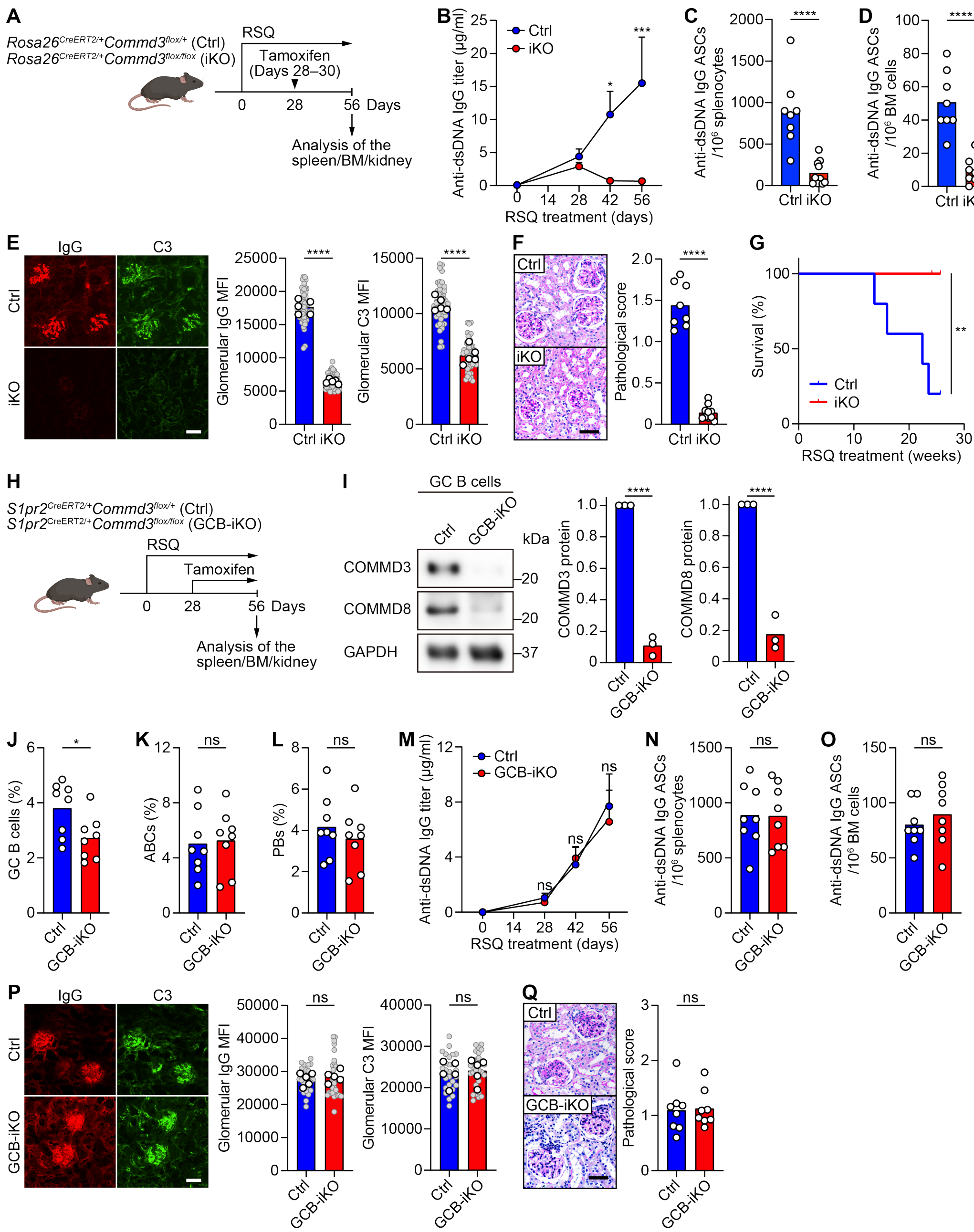

Supplemental Figure 5. Effects of systemic or GC B cell-specific COMMD3/8 complex deficiency on lupus pathology. (**A**) Experimental diagram for (B) to (G). *Rosa26*$^{CreERT2/+}$*Commd3*$^{flox/+}$ (Ctrl) and *Rosa26*$^{CreERT2/+}$*Commd3*$^{flox/flox}$ (iKO) mice were treated with RSQ and administered tamoxifen. (**B**) Serum anti-dsDNA IgG titers analyzed by ELISA. Data are shown as mean + SEM (*n* = 8 Ctrl, 10 iKO). (**C** and **D**) Anti-dsDNA IgG ASC numbers in the spleen (C) and BM (D) analyzed by ELISPOT. (**E**) IgG and C3 deposits in the renal glomeruli analyzed by immunofluorescence microscopy. MFI of IgG and C3 was quantified. (**F**) PAS-stained glomerular sections and pathological scores. (**G**) Survival rates of Ctrl (*n* = 5) and iKO (*n* = 7) mice. (**H**) Experimental diagram for (I) to (Q). *S1pr2*$^{CreERT2/+}$*Commd3*$^{flox/+}$ (Ctrl) and

S1pr2$^{CreERT2/+}$*Commd3*$^{flox/flox}$ (GCB-iKO) mice were treated with RSQ and administered tamoxifen. (**I**) Immunoblot analysis of COMMD3 and COMMD8 protein levels in GC B cells from the spleens of Ctrl and GCB-iKO mice on day 56 of RSQ treatment. Representative blots and quantification of COMMD3 and COMMD8 protein levels normalized to GAPDH are shown. (**J**–**L**) Frequencies of GC B cells (J), ABCs (K), and PBs (L) in the spleen analyzed by flow cytometry. (**M**) Serum anti-dsDNA IgG titers analyzed by ELISA. Data are shown as mean + SEM (n = 8). (**N** and **O**) Anti-dsDNA IgG ASC numbers in the spleen (N) and BM (O) analyzed by ELISPOT. (**P**) IgG and C3 deposits in the renal glomeruli analyzed by immunofluorescence microscopy. MFI of IgG and C3 was quantified. (**Q**) PAS-stained glomerular sections (left) and pathological scores (right). Data are shown as means, with symbols representing individual mice (C–F, I–L, and N–Q). In (E) and (P), gray symbols represent individual glomeruli. In (E), (F), (P), and (Q), representative images are shown (scale bar, 50 μm). Results are representative of two independent experiments. P values were determined by two-way ANOVA with Sidak's post hoc test (B and M), two-tailed unpaired t test (C–F, I–L, and N–Q), or log-rank test (G). *$P < 0.05$; **$P < 0.01$; ***$P < 0.001$; ****$P < 0.0001$; ns, not significant.

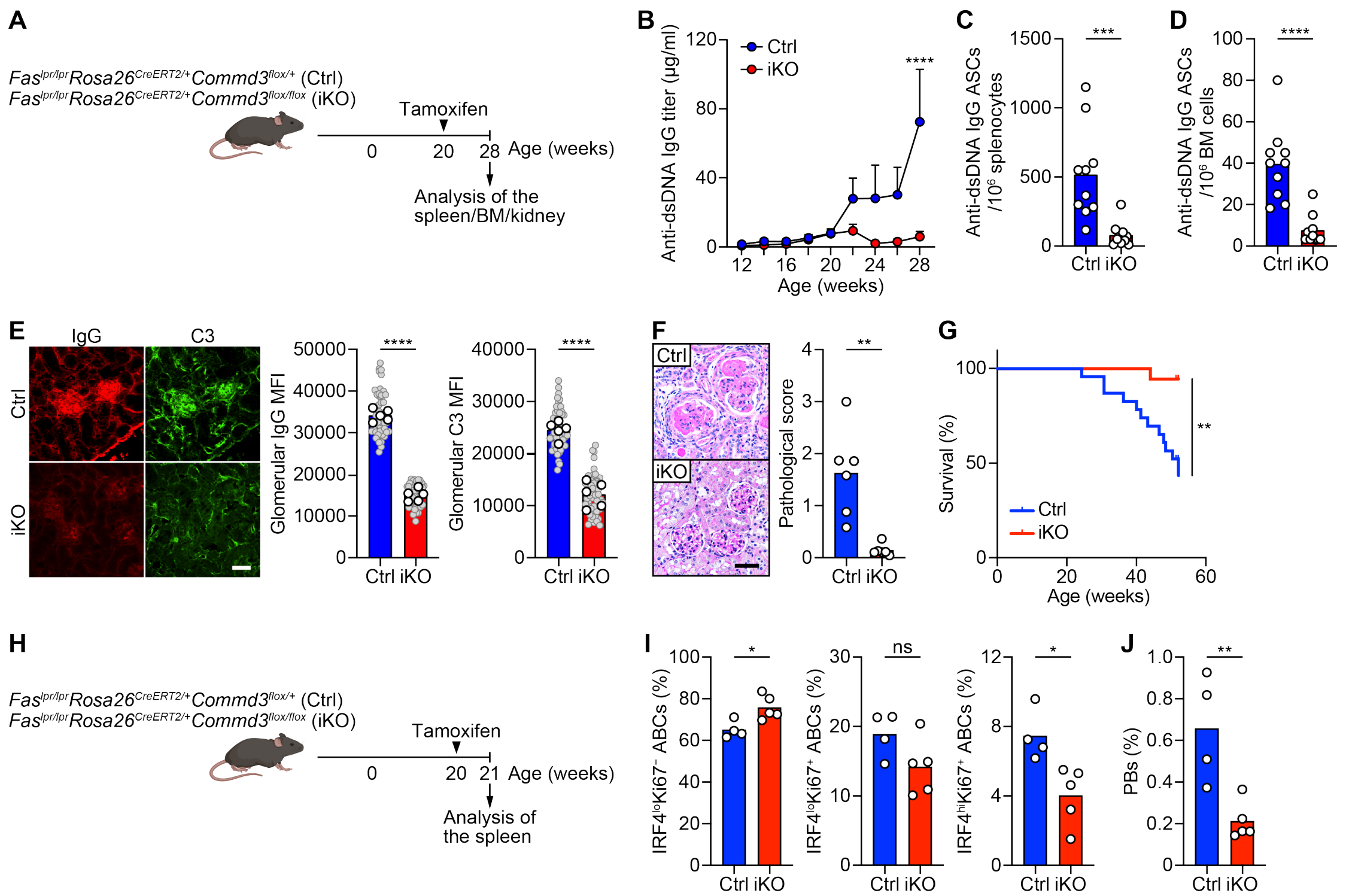

Supplemental Figure 6. Effects of COMMD3/8 complex deficiency on spontaneous lupus. (**A**) Experimental diagram for (B) to (G). *Fas*$^{lpr/lpr}$*Rosa26*$^{CreERT2/+}$*Commd3*$^{flox/+}$ (Ctrl) and *Fas*$^{lpr/lpr}$*Rosa26*$^{CreERT2/+}$*Commd3*$^{flox/flox}$ (iKO) mice were administered tamoxifen. (**B**) Serum anti-dsDNA IgG titers analyzed by ELISA. Data are shown as mean + SEM (n = 10). (**C** and **D**) Anti-dsDNA IgG ASC numbers in the spleen (C) and BM (D) analyzed by ELISPOT. (**E**) IgG and C3 deposits in the renal glomeruli analyzed by immunofluorescence microscopy. MFI of IgG and C3 was quantified. (**F**) PAS-stained glomerular sections and pathological scores. (**G**) Survival rates of Ctrl (n = 23) and iKO (n = 18) mice. (**H**) Experimental diagram for (I) and (J). Ctrl and iKO mice were treated with tamoxifen, and splenocytes were analyzed by flow cytometry. (**I** and **J**) Frequencies of ABC populations in total ABCs (I) and of PBs in splenocytes (J). Data are shown as means, with symbols representing individual mice (C–F, I, and J). In (E), gray symbols represent individual glomeruli. In (E) and (F), representative images are shown (scale bar, 50 μm). Results are representative of two independent experiments. P values were determined by two-way ANOVA with Sidak's post hoc test (B), two-tailed unpaired t test (C–F, I, and J), or log-rank test (G). *$P < 0.05$; **$P < 0.01$; ***$P < 0.001$; ****$P < 0.0001$; ns, not significant.

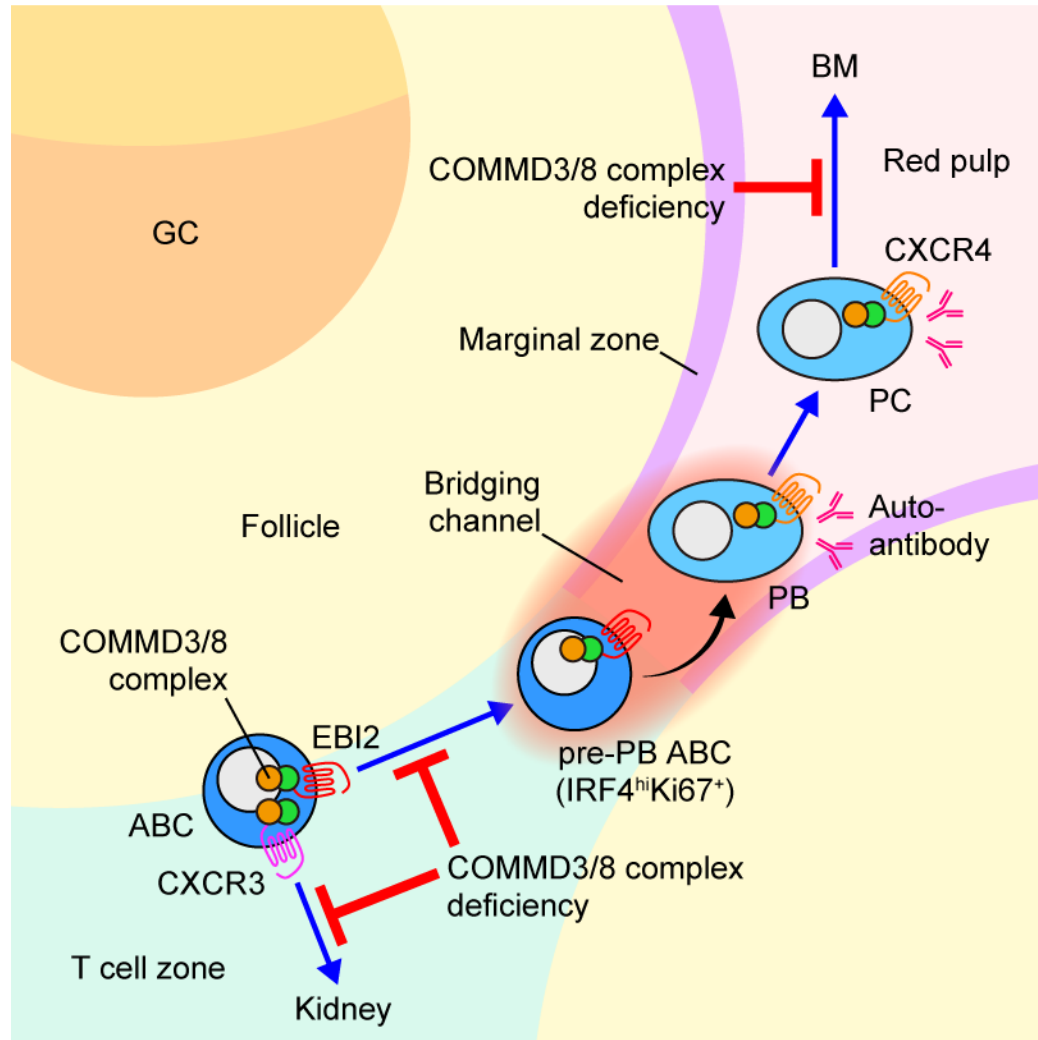

Supplemental Figure 7. Migration-dependent program driving EF differentiation of ABCs into autoreactive PBs. During EF responses in lupus, ABCs give rise to autoreactive PBs via a pre-PB ABC state (IRF4^{hi}Ki67^{+}). This process depends on EBI2-mediated migration of ABCs to the bridging channels of the spleen. The COMMD3/8 complex is required for multiple migratory processes in pathogenic B-lineage cells, including ABC positioning in the bridging channels, ASC homing to the BM, and ABC infiltration into the kidney. Deficiency of the COMMD3/8 complex impairs these migratory processes and ameliorates lupus pathology.

Supplemental Table 1. Clinical characteristics of a treatment-naïve patient with SLE.

Parameter (unit) [reference range]	Result
Ancestry	Japanese
Age (years)	22
Sex	Female
Disease duration (months)	12
WBC count (10^3/μl) [3.3–8.6]	4.4
RBC count (10^6/μl) [3.86–4.92]	3.11
Hemoglobin concentration (g/dl) [11.6–14.8]	7.8
Platelet count (10^3/μl) [158–348]	323
C3 (mg/dl) [73–138]	83
C4 (mg/dl) [11–31]	8.7
50% hemolytic complement activity (U/ml) [25–51]	25.7
IgA (mg/dl) [93–393]	180
IgM (mg/dl) [50–269]	192
IgG (mg/dl) [861–1747]	2724
Antinuclear antibody [< 1:40]	1:2560
Anti-dsDNA IgG (IU/ml) [< 12]	25
Anti-Sm antibody (U/ml) [< 10]	600
Anti-U1RNP antibody (U/ml) [< 10]	512
Anti-cardiolipin antibody (U/ml) [< 20]	47.7
Lupus anticoagulant [< 1.2]	1.1
Anti-β2-glycoprotein I antibody (U/ml) [< 20]	18.9
Urine protein (g/gCre)	3.21
Fever	Yes
Hematologic features (leukopenia, thrombocytopenia, autoimmune hemolysis)	No
Neuropsychiatric features (delirium, psychosis, seizure)	No
Mucocutaneous features (alopecia, cutaneous lupus, oral ulcers)	Yes
Serositis	No
Arthritis	Yes
Nephritis	Yes (Class IIIA + V)
SLEDAI	27
Treatment	Naïve
Overlap connective tissue diseases	No

Supplemental Table 2. Demographics of SLE patients.

Parameter (unit)	SLE (*n* = 60)
Ancestry (% Japanese)	100
Age (years)	44 (16–76)*
Sex (% female)	87
Disease duration (months)	179 (1–588)*
C3 (mg/dl)	93 (16–140)*
C4 (mg/dl)	20 (1.4–97)*
IgG (mg/dl)	1585 (528–4356)*
Anti-dsDNA IgG (IU/ml)	31 (2–200)*
SLEDAI	3 (0–18)*
Treatment (%)	
Naïve	8
Prednisolone	75
Hydroxychloroquine	60
Mycophenolate mofetil	37
Tacrolimus	25
Belimumab	22
Azathioprine	10
Anifrolumab	5

*Mean (range)
