# Supplementary material for: Migration-dependent extrafollicular programming of preplasmablast age-associated B cells drives lupus pathogenesis
Source: J Clin Invest. 2026 Jul 14;136(16):e204095. doi: 10.1172/JCI204095 (PMC13476141; doi:10.1172/JCI204095)

Full unedited blots for Supplemental Figure 4A

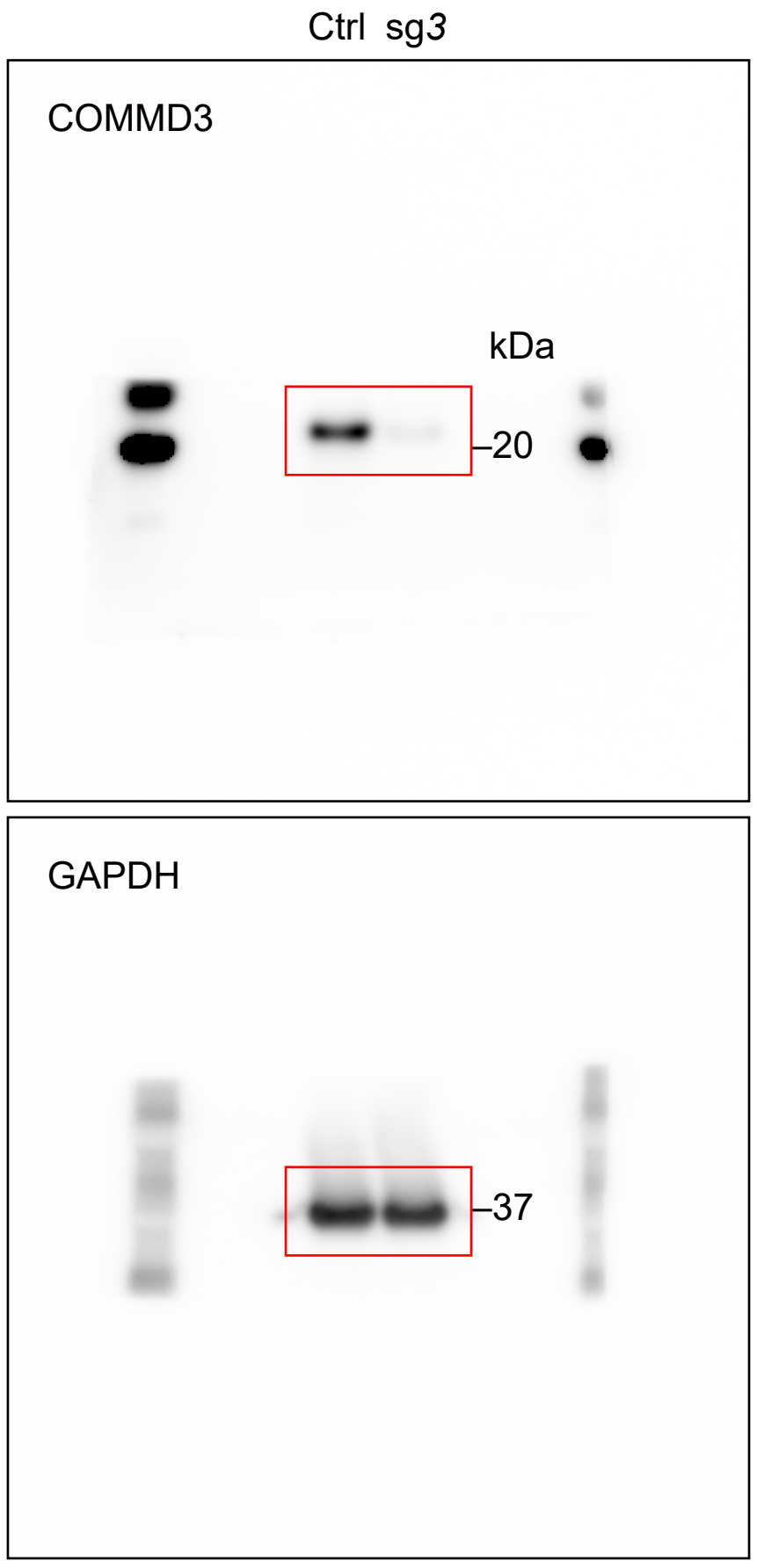

Full unedited blots for Supplemental Figure 5I

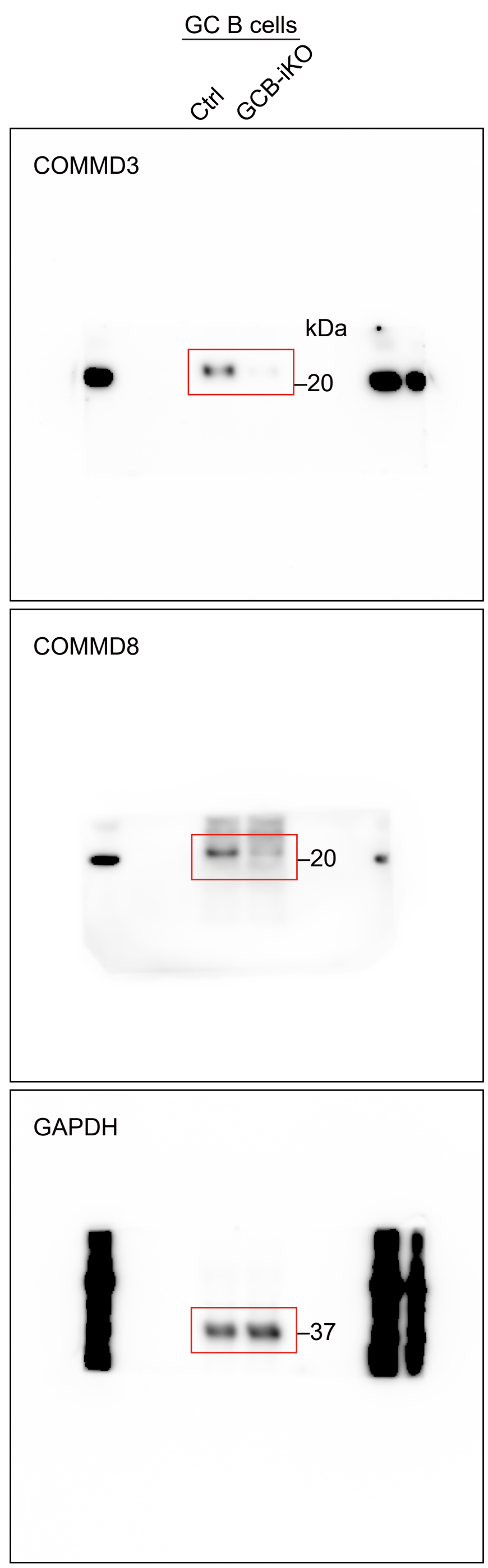

Supplement: Supplementary file 2 [file jci-136-204095-s039.pdf]
